# Wave dynamics alteration by discontinuous flexible mats of artificial seagrass can support seagrass restoration efforts

**DOI:** 10.1038/s41598-023-46612-z

**Published:** 2023-11-08

**Authors:** Raúl Villanueva, Maike Paul, Torsten Schlurmann

**Affiliations:** Ludwig Franzius Institute for Hydraulic, Estuarine and Coastal Engineering, Leibniz University Hannover, 30167 Hannover, Germany

**Keywords:** Ecology, Environmental sciences, Ocean sciences, Engineering

## Abstract

Seagrass restoration can be promoted through the use of artificial seagrass (ASG). However, there is no guideline for ASG design, which requires a sound understanding of the inherent hydrodynamics in a submerged environment. Present know-how primarily stems from idealized ASG attached to a fixed bed. To develop accessible field deployment for restoration, anchored prototype scale ASG mats (coconut mesh) were proposed and tested under differing wave conditions. The aim of this study was then to: 1) analyze hydrodynamic interaction of ASG mats; and 2) assess the suitability of contemporary predictive hydrodynamic models. Velocity structure and wave propagation were measured around one and two ASG mats (separated by a 2-m gap). The mats reduced orbital velocities by up to 16% (2 mats), whereby the average reduction of all tested vegetated conditions was low ($<10\%$) compared to the non-vegetated conditions. Velocities increased above the ASG, with the gap enhancing velocity (up to 11%) instead of attenuating it. Wave decay followed an exponential decrease, further enhanced by the second mat. Current models did not capture the induced hydrodynamics for the full range of wave conditions tested, with the second mat increasing uncertainties. Wave decay models generally overestimated wave attenuation (up to 30%), except for longer wave periods. Nevertheless, for the full range of conditions, the models provide accurate insight into the expected magnitude of attenuation under field conditions. It is speculated that mat flexibility affects the surrounding hydrodynamics through inherent motion, with the gap contributing to the uncertainties.

## Introduction

Seagrass meadows have experienced great losses in recent decades, mostly due to human development^1,2^. They represent important coastal ecosystems that provide services to the environment and to human populations^3^, including fish habitat, livelihood for coastal communities, and carbon sequestration^4^. Seagrasses alter their environment drastically by reducing wave energy and current velocity^5^, lowering turbidity by increasing sedimentation^6,7^, and stabilizing the coastline by reducing erosion^8–10^. This makes these ecosystems important actors in coastal protection schemes and priority targets in conservation and restoration within the contemporary key concept of nature-based solutions^11,12^.

Several methods of seagrass restoration exist^13^, with no go-to method unanimously considered the most efficient. Single-shoot transplantation techniques, for example, have been shown to be successful^14^; however, they can also be expensive and time intensive. A widely accepted notion is that seagrasses provide themselves with the means of survival through positive feedback mechanisms^15^. From an ecosystem engineering perspective, this means that seagrasses modify their local environmental dynamics (e.g. flow-induced energy flux and mass transport) in such a way that ensures survival and promotes further proliferation. Preliminarily, this can be externally achieved through shelter provided by other structures^16^. Building on this, Carus et al.^17^ proposed the use of biodegradable mats of artificial seagrass (ASG) to serve as shelter for real seagrass and thus promote growth. ASG would then provide the protection that spawning seedlings need by emulating seagrass coastal protection services.

The proposed mats must be anchored to the seabed as they undergo hydrodynamic loading. Considering the inherent costs of field applications, these anchors should be discrete (i.e. a fixed number of punctual anchors), while able to resist the highest expected hydrodynamic loads. Further, to adapt to the highly dynamic environment, both mats and ASG should be flexible. Usage of flexible mats in the context of restoration makes sense, as these can be manufactured extrinsically, easily transported and deployed in the field; however, this flexibility also means that their interaction with the surrounding environment is complex. Presently, most understanding of flow-vegetation interaction focuses on shoots fixed to rigid, non-mobile base layers. Nonetheless, the importance of plant flexibility was rapidly recognized as flow-vegetation interaction research began, with concepts like relative plant velocity^18^ (plant sway with water particle motion reduces drag, thus reducing flow attenuation) and the *monami* phenomenon (coherent motion of the meadow alters the in-canopy and wake velocity structure, see e.g.^19^) showing that flexibility should not be neglected. A flexible mat then means that the whole meadow comes into motion, limited only by the amount of anchoring points used.

To date, other than scour protection studies with commercial motivation^20^, there is no research regarding flexible, discretely anchored mats under marine conditions. Moreover, current predictive models of flow-vegetation interaction have been validated for either idealized laboratory conditions^21,22^ (i.e. fixed vegetation and uniformly anchored rigid base layers), or natural vegetation in the field^23^. The experiments described in this study revolved around the applicability of state-of-the-art flow-vegetation models to predict wave dynamics around flexible mats under oscillatory flow conditions. The loads undertaken by the anchoring points was investigated by Villanueva et al.^20^. Interestingly, the study found that about 95% of wave-induced drag on the flexible ASG mats could be explained by existing drag formulations based on meadow morphology and incident hydrodynamics. A similar effect may then be expected for both the velocity structure along the water column and the wave height evolution along the meadow. Specifically, we a) analyze the effect of flexible ASG, discretely anchored to a sand bed via a flexible base layer, on wave-induced flow velocities and wave propagation; b) compare this with the status quo of wave-vegetation interaction research; and c) discuss the suitability of currently accepted models applied to fully flexible anchored mats intended for field applications.

## Theoretical background

The interaction between submerged vegetation and hydrodynamics has been widely studied, with a great deal of focus given to unidirectional flow^24–27^. Interest in submerged macrophytes and their effect on oscillatory flow, i.e. waves, gained momentum with the concept of energy dissipation^28^. It was noted that wave decay took place in areas where either changes in morphology were visible, or where submerged vegetation were present. It became clear that the latter have a complex interaction with hydrodynamics, partly observable through wave decay, but also penetrating the water column and affecting the velocity structure of the oscillatory (orbital) flow. Nevertheless, despite increased research surrounding this interaction, predictive methods to describe wave decay and oscillatory flow attenuation tend to be set-up-specific, hence delivering differing and even conflicting results. Supplementary Table S1 presents a summary of relevant studies applying different methodologies to investigate wave-vegetation interaction. With different target parameters, the obtained results and conclusions can vary greatly, even for similar input conditions and experimental set-ups.

### Wave decay

Wave energy dissipation has predominantly been the starting point for any formulation of wave decay^28^. The energy dissipation formulation is based on the steady conservation of energy flux^29^ $\partial Ec_g/\partial x=-\varepsilon _D$, where $E=(1/8)\rho gH^{2}$ is the energy density, $\rho$ the water density, *g* the gravitational constant, *H* the wave height, $c_g$ the group velocity, *x* the distance within the vegetation, and $\varepsilon _D$ is the vegetation-induced rate of energy dissipation. Water waves propagating through submerged and emergent vegetation lose energy by performing work on the vegetation stems, which directly results in smaller wave heights^28^.

Based on bottom interaction, wave decay was considered to be exponential through the wave decay ratio $H=H_0\exp (-k_ix)$, based on the real part of the complex wave number ($k=k_r-ik_i$) and calculated through the solution to the dispersion relation^30^. However, for vegetated areas, a non-exponential wave decay was also proposed, with the following equation still widely used today^28^:

1 $$\begin{aligned} H(x) = \frac{H_0}{1+\beta x} \end{aligned}$$

where $H_0$ is the incident wave height at the leading edge of the meadow, *H*(*x*) is the wave height at distance *x* from the leading edge and in the direction of wave propagation, and $\beta$ is the so-called damping coefficient. $\beta$ has been modified extensively from its original proposition^28^ depending on different boundary conditions, but can be expressed in general terms for flexible meadows through Eq. (2):

2 $$\begin{aligned} \beta =\frac{4}{9\pi }C_Db_vNH_0k\frac{\sinh ^3kl_{e}+3\sinh kl_e}{(\sinh {2kd}+2kd)\sinh {kd}} \end{aligned}$$

where *N* is the shoot areal density, $b_v$ the plant width, $C_D$ the drag coefficient, *d* the water depth and $l_e$ the vegetation effective length. $l_e$ refers to the upright length of a rigid meadow for which a flexible meadow of canopy height $h_c$ will exert an equivalent force on flow^31,32^. This occurs due to reconfiguration of the plant caused by flow (steady or unsteady), such that the height of the meadow becomes lower than $h_c$, in turn reducing drag. Losada et al.^33^ extended the original formulation^28^ using the reconfigured meadow height instead of the upright length, successfully predicting decay within flexible canopies. This length, however, needs to be actively measured, which means more complicated set-ups, especially for field applications. The use of $l_e$ allows for the implementation of the model for flexible canopies without the need to measure the actual plant reconfiguration.

Note that Eq. (2) makes use of the drag coefficient $C_D$ and the geometric and spatial properties of the dissipating mechanism (i.e. submerged vegetation). Boundary reflection (e.g. end of a flume in a laboratory) and morphological features cause modulation of waves so that in any particular scenario, $C_D$ needs to be calibrated through experimentation^29^. Nonetheless, the calibrated $C_D$ will still vary greatly depending on a myriad of factors, such as load type and intensity (pure waves vs. currents vs. a combination of both), plant flexibility and morphological characteristics, and whether plant motion is taken into account^34^. Experimentally calibrated values have been commonly related to either the Reynolds Number ($Re=ub/\nu$, with *u* the velocity, $\nu$ the kinematic viscosity, and *b* the characteristic length) or the Keulegan-Carpenter Number ($KC=uT/b$, with *T* the wave period), with studies showing that the latter is well suited for low-energy (inertia-dominated) conditions and the former for turbulent areas^35–37^. Experimental calibration of $C_D$ based on *Re* has been commonly given in general terms by:

3 $$\begin{aligned} C_D=n_1+\left( \frac{n_2}{Re}\right) ^{n_3} \end{aligned}$$

where $n_i$ are constants that depend on the fitted experimental conditions. Notice, however, that the range of validity of *Re* for any fitted case may vary depending on the trialed conditions^38,39^. Initially, values of $C_D$ between 0.1–1 were employed based on the value for a rigid cylinder; nevertheless, the inclusion of vegetation motion^18^ and the use of in-canopy velocities to calculate *Re*^21^ have led to a common use of values between 2–3, reaching up to *O*(2) for low *Re*. *Re* may also differ depending on the chosen input parameters, with arguments for the use of top-of-canopy velocity against in-canopy, as the latter is impractical to measure^33^, and using $l_e$ to incorporate flexibility in the calibration of $C_D$:

4 $$\begin{aligned} Re^{l_e}=\frac{ul_e}{\nu } \end{aligned}$$

Regarding calibration based on *KC*, a formulation of $C_D$ based on the calibration for flat plates was proposed and validated for application with flexible submerged vegetation under both unidirectional currents and oscillatory flow^22^:

5 $$\begin{aligned} C_D=\textrm{max}(10KC^{-\frac{1}{3}},1.95) \end{aligned}$$

where the minimum threshold of 1.95 was found to accurately describe drag induced by flexible blades for the unidirectional limit. Eq. (5) has been successfully applied to calculate drag forces on anchors under pure wave conditions^20^ and wave damping under combined waves and currents^40^. Other *KC*-based formulations of $C_D$ following the form of Eq. (3) have been successfully validated to obtain a bulk $C_D$ for regular and irregular waves with model and real vegetation^36^.

### Oscillatory flow

The velocity structure within the water column can be decomposed into three parts^21^: steady flow, oscillatory or time-varying, and turbulent. The instantaneous velocity at an arbitrary point in space and time can then be written as:

6 $$\begin{aligned} U_i(z,t)=U_c(z)+U_w(z,t)+U'(z,t) \end{aligned}$$

where $U_i$ is the instantaneous velocity, $U_c$ the steady flow component, $U_w$ the oscillatory component and $U'$ the turbulent component. The steady current $U_c$ observed under oscillatory flow was originally related to the mass transport velocity and solved analytically in terms of Stoke’s Stream Function^41^. Non-linearity of the oscillatory flow, caused in part by near-bed viscosity, can be captured through higher order solutions. This non-linearity means that the vertical and horizontal components of 2D wave motion are not 90$^{\circ }$ out of phase, as suggested by linear wave theory, resulting in a non-zero wave stress analogous to the turbulent Reynolds stress^31^. The mass transport velocity (here $U_c$) of a progressive wave within the water column has been solved analytically by solving the stream function^41^ (Eq. (7)). Eq. (7) is valid for small values of mass transport velocity compared to the orbital velocity.

7 $$\begin{aligned} U_c= & {} \frac{a^2\omega k}{4\sinh ^2{kd}}\Biggl [2\cosh {\left( 2kd\left( \frac{z}{d}-1\right) \right) }+3\nonumber \\{} & {} +kd\sinh {2kd}\left( 3\left( \frac{z}{d}\right) ^2-4\left( \frac{z}{d}\right) +1 \right) +3\left( \frac{\sinh {2kd}}{2kd}+\frac{3}{2}\right) \left( \left( \frac{z}{d}\right) ^2-1\right) \Biggr ] \end{aligned}$$

where *a* is the wave amplitude, $\omega$ the wave angular frequency, and *z* the height from the bed.

The oscillatory component of the velocity is more complex due to the cyclic nature of motion, where inertia plays an important role. Linear and higher order wave theories can predict these oscillatory velocities accurately; however, flow-vegetation interaction additionally requires: (1) an understanding of the geometric properties of the vegetation, given by the vegetation element frontal $\lambda _f$ and planar $\lambda _p$ proportion to the surface area:

8a $$\begin{aligned} \lambda _f&=\frac{h_cb_v}{S^2} \end{aligned}$$

8b $$\begin{aligned} \lambda _p&=\frac{b_vt_v}{S^2} \end{aligned}$$

where *S* is the average separation between shoot central axes in both planar directions (*x*-*y*), and $t_v$ is the plant thickness; and (2) a scaling of these geometric properties with respect to the hydrodynamic environment, given by the canopy shear length scale $L_s$ and the drag length scale $L_d$^21^:

9a $$\begin{aligned} L_s&=\frac{2h_c}{C_f} \end{aligned}$$

9b $$\begin{aligned} L_d=&\frac{2h_c(1-\lambda _p)}{C_D\lambda _f} \end{aligned}$$

where $C_f$ is the friction coefficient. Lowe et al.^21^ proposed an analytical model to estimate flow within a canopy based on incident hydrodynamics and canopy geometric properties. The model balances the acceleration and force terms given by the set of Eqs. (8) and (9) and the oscillatory velocity $U_w$. They then developed a non-dimensionalized form of the model in terms of the wave orbital excursion $A_{\infty }^{\textrm{rms}}$ to determine the relative magnitude of each of the terms:

10 $$\frac{{\partial (\hat{U}_{w}^{*} - U_{{\infty ,w}}^{*} )}}{{\partial t^{*} }} = \frac{{A_{\infty }^{{{\text{rms}}}} }}{{L_{s} }}|U_{{\infty ,w}}^{*} |U_{{\infty ,w}}^{*} - \frac{{A_{\infty }^{{{\text{rms}}}} }}{{L_{d} }}|\hat{U}_{w}^{*} |\hat{U}_{w}^{*} - \frac{{C_{M} \lambda _{p} }}{{1 - \lambda _{p} }}\frac{{\partial \hat{U}_{w}^{*} }}{{\partial t^{*} }}$$

11 $$\begin{aligned}{} & {} A_\infty ^{\textrm{rms}}=\frac{\hat{U}_{\infty ,w}^{\textrm{rms}}}{\omega } \end{aligned}$$

where $C_M$ is the inertia coefficient, and $A_{\infty }^{\textrm{rms}}$ is based on the root mean square free stream velocity $U_{\infty ,w}^{\textrm{rms}}$ (canopy unaffected). The asterisk in Eq. (10) indicates the velocity and time parameters non-dimensionalized through their product with $(U_{\infty ,w}^{\textrm{rms}})^{-1}$ and $\omega$, respectively. The over-hat represents the canopy-integrated values ($z=[0:h_c]$). The attenuation of in-canopy oscillatory velocity was then set as the ratio of canopy-averaged root mean square wave velocity and the corresponding free stream velocity:

12 $$\begin{aligned} \alpha _w=\frac{\hat{U}_{w}^{\textrm{rms}}}{\hat{U}_{\infty ,w}^{\textrm{rms}}} \end{aligned}$$

The dimensionless terms in Eq. (10), i.e. $A_{\infty }^{\textrm{rms}}/L_s$, $A_{\infty }^{\textrm{rms}}/L_d$ and $C_M\lambda _p/(1-\lambda _p)$, are of *O*(1), whence the relative importance of each term is determined. Depending on the hydrodynamic conditions, $\alpha _w$ can be dominated by one or more of these terms, which can then reduce the solution of Eq. (10) to disambiguate different flow conditions^21^: canopy-independent ($\alpha _w=1$), inertia-dominated ($\alpha _w=(1-\lambda _p)/(1+(C_M-1)\lambda _p)$), general flow ($\alpha _w(A_{\infty }^{\textrm{rms}}/L_s,A_{\infty }^{\textrm{rms}}/L_d,C_M\lambda _p/(1-\lambda _p))$), and unidirectional limit or current-dominated ($\alpha _w=\sqrt{L_d/L_s}$).

## Methodology

To study the response of flexible mats of artificial seagrass in a marine environment, a series of experiments were carried out under controlled laboratory conditions. Initially, different mats and anchor configurations were tested, with mat mechanical performance, flow interaction, and the resulting loads on anchoring points scrutinized^20^. Here, we focus on the velocity structure and wave height evolution. The compound set of experiments was carried out at the Schneiderberg Wave Flume (WKS) at Ludwig Franzius Institute of Leibniz University Hannover. The WKS is a large-scale wave flume with a length of 110 m, a width of 2.2 m, and a depth of 2 m. Waves are generated by an electrical paddle-type wave-maker with a maximum paddle stroke of 1.8 m and wave height generation of up to 0.5 m.

Most flow-vegetation physical experiments are carried out in scaled-down form for practical reasons, with wave heights $<20$ cm and periods $<2$ s (Supplementary Table S1). Here, the large scale of the flume allowed us to test prototype-sized mats^17,20^, which provides insight into the potential hydrodynamic response of the mats under field conditions. Typical near-bed velocities found around seagrass-prone areas are usually lower than 0.5 m s$^{-1}$^42,43^; consequently, velocities within this range were sought and input wave conditions selected by calculating the expected near-bed velocity based on linear wave theory. This resulted in wave periods from 2–5 s and wave heights up to 33 cm. Three different water depths were selected, leading to 12 different wave conditions (Table 1). Note that the near-bed velocities in Table 1 were used as estimates of the expected velocities, and are given only as reference. The true nonlinear velocities were measured and are presented within the results. Further, to support the transmission of these conditions to other laboratory and field experiments, the dimensionless parameters *kd* and Ursell Number ($U_R=H\lambda ^2/d^3$, where $\lambda$ is the wavelength) are also given in Table 1. The tested wave trains consisted of 60 regular waves for each of the conditions tested.

**Table 1:** Wave conditions tested for each wave run (WR). *Expected values of $\lambda$ and the maximum horizontal orbital velocity 3 cm above the bed $U_b^{\textrm{max}}$ were calculated based on linear wave theory for reference.

Parameter		WR1	WR2	WR3	WR4	WR5	WR6	WR7	WR8	WR9	WR10	WR11	WR12
*T*	[s]	2.00	3.00	4.00	5.00	2.00	3.00	4.00	5.00	2.00	3.00	4.00	5.00
*H*	[m]	0.11	0.06	0.19	0.11	0.22	0.11	0.06	0.22	0.33	0.22	0.11	0.06
*d*	[m]	0.50	0.50	0.50	0.50	0.63	0.63	0.63	0.63	0.83	0.83	0.83	0.83
$\lambda$	[m]*	4.06	6.40	8.67	10.92	4.44	7.09	9.66	12.19	4.92	8.04	11.03	13.97
$h_c/d$	[-]	0.50	0.50	0.50	0.50	0.40	0.40	0.40	0.40	0.30	0.30	0.30	0.30
$U_b^{\textrm{max}}$	[m s$^{-1}$]*	0.17	0.11	0.36	0.21	0.30	0.18	0.10	0.38	0.36	0.30	0.16	0.09
$U_R$	[-]	14.48	19.64	114.33	105.03	17.57	22.44	22.71	132.66	13.80	24.61	23.17	20.27
*kd*	[-]	0.77	0.49	0.36	0.29	0.89	0.56	0.41	0.32	1.06	0.65	0.47	0.37

### Experimental set-up

**Figure 1:**
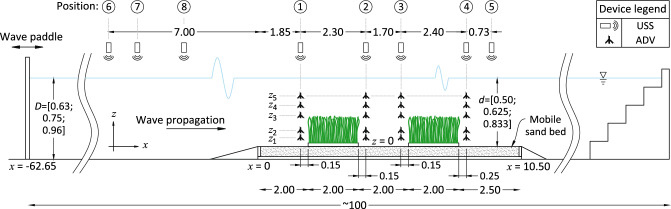
Schematic of the experimental set-up used for the experiments. *D* = water depth to the concrete flume bed; *d* = depth to sand bed. Shown are the device positions. USS = Ultrasonic Sensor; ADV = Acoustic Doppler Velocimeter. $z_i$ are the different ADV positions, where each of the 4 ADVs was vertically displaced to the *i* positions labeled, with $z_{1-5}=[3.3; 10.5; 25.7; 35.9; 45.7]$ [cm]. *z*-axis exaggerated by 4 times. All dimensions in meters.

A mobile sand bed (homogeneously graded quartz of $d_{50}=0.19$ mm, particle density of 2.65 g cm$^{-3}$, and bulk density of 1.45 t m$^{-3}$) was constructed on top of the concrete bed of the flume. The sand bed began 62.65 m from the idle paddle position, had a length of 10.5 m, a width equal to that of the flume, and a depth of 10 cm (Fig. 1). 2.7 cm-thick plywood panels were installed below the sand bed to facilitate instrument and anchor mounting. The sand bed was preceded by a 1:30 plywood ramp and succeeded by a 1:10 gravel ramp. The far end of the flume was equipped with an artificial beach consisting of an aluminum stepped slope and industrial foam to enhance wave absorption. $x=0$ was aligned with the beginning of the sand bed; the *x*-axis ran parallel to the flume and was positive in the direction of wave propagation, the *y*-axis ran the cross-section with 0 at the right flume wall (with respect to the direction of wave propagation), and the *z*-axis along the vertical with 0 at the sand bed.

Prototype ASG mats were built to simulate mats that could be deployed in the field for restoration purposes^17^. For this, 2x2-m mats were assembled using rolls of coconut mesh of different compositions^20^. Individual ASG stems were then fixed to this hybrid coconut mat to create a fully flexible ASG mat. Polyamide cable ties (PA, density $\delta _{\textrm{PA}}=1.13$ g cm$^{-3}$ and flexural rigidity $EI_{\textrm{PA}}=800$ N mm$^{2}$) of length $h_c=250$ mm, width $b_v=4.8$ mm, and thickness $t_v=1.36$ mm were used as a seagrass surrogate. Density effects were not a focal point of this study, therefore, a constant shoot density of $N=400$ m$^{-2}$ was chosen, which resulted in a shoot separation of $S=5$ cm and a frontal area per canopy volume of 1.92 m$^{-1}$ ($b_v/S^2$)^44^. The chosen ASG had low flexibility and the meadow density was kept low compared to real meadows to test the hydrodynamic effect of a stiffer, sparse meadow. Nonetheless, it has been shown that PA reconfigures with hydrodynamic loading while simultaneously affecting the flow field around a single stem^45^. The variation of depth resulted in different submergence ratios $h_c/d=[0.3,0.4,0.5]$ (Table 1).

To quantify wave decay, a total of 8 Ultralab Ultrasonic Sensors (USS) with a resolution of 0.2 mm were used to measure the water level fluctuations $\eta (t)$ at different positions along the *x*-axis (Fig. 1). The position of the devices are enumerated (starting with the sand bed), with positions 1–4 placed immediately in front of and immediately behind the ASG mats to directly measure the effect of the mats on wave height evolution and velocity structure. Position 5 (last USS in *x*) was located 1.52 m before the end of the sand bed. Three USS were positioned in front of the sand bed as control points (positions 6–8) for the input wave conditions (Table 1), with positions 7 and 8 varying depending on the wave condition used to cover different points along one wavelength. An HBM signal amplifier bundled with the software Catman was used to synchronously measure water level fluctuations, pressure, and forces at a frequency of 100 Hz. Wave orbital velocities were recorded simultaneously by means of a trigger connected to the HBM. Four downward-looking Nortek Vectrino+ Acoustic Doppler Velocimeters (ADV) were used to measure the orbital velocities at 50 Hz. The *u*, *v*, and *w* components of the measured velocities were respectively aligned with the *x*, *y*, and *z* components of the set-up. Throughout this paper, specific devices and their position will be referred to by the given acronym directly followed by the position number (e.g. ADV1 corresponds to the ADV position 1, in front of the first ASG mat, Fig. 1). The four ADVs were aligned in *z* with each other and in *x* with USS1–USS4.

For each wave run (WR), the 8 USS and 4 ADVs recorded simultaneously. To build the orbital velocity profiles, the ADVs were displaced vertically ($z_i$ in Fig. 1) and the measurements repeated for the corresponding WR. Finally, this was repeated for three different cases: 1) no vegetation, as control; 2) under the presence of 1 ASG mat, starting at $x=2$ m; and 3) with 2 ASG mats with a 2-m gap between them. This resulted in a total of 168 wave run measurements.

### Data processing and analysis

All recorded data were imported and processed in matlab (R2022a). Water level fluctuations for all USS data were standardized (mean = 0) around the still water level using the detrend function of matlab. The original time series was curated by removing the initial incoming and outgoing waves corresponding to the wave generator ramp time. Self-cross-correlation and a Fast Fourier Transform were used to calculate the period *T* of the incoming waves over the sand bed. The calculated *T* did not vary from the input *T* (Table 1) by more than 2%; therefore, from here on, the tabulated input values are used for simplicity. Zero-up-crossing was used to identify the first full wave of the curated time series, after which 10 waves were extracted to obtain a time series with a length equal to 10*T*. This was done to decrease the effect of wave reflection from the far end of the flume. The 10-wave time series window was then averaged into a single representative wave for the respective wave run using a phase averaging technique^46^. The maximum and minimum values of $\eta (t)$ for each wave run were then calculated from the phase-averaged wave. Table 2 shows the measured wave heights at the leading edge ($H_0$) and the ratio $H/H_0$ for positions 2 and 4, i.e. just behind each ASG mat.

**Figure 2:**
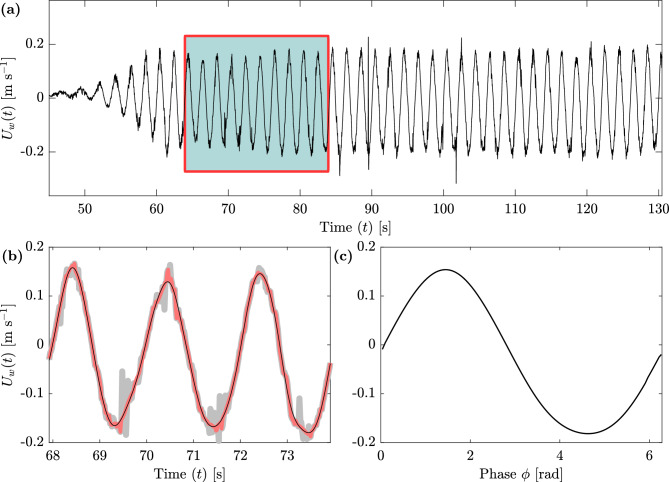
Processing of the orbital velocity time series for WR1, no artificial vegetation, ADV position 4 (see Fig. 1 and Table 1). (a) shows the raw data record of the ADV with the 10-wave window that was extracted for post-processing. (b) shows a 3-wave window comparing the raw data (gray, thick line), the despiked data after^47^ (red, medium thick line), and the filtered data (thin, solid black line). (c) shows the resulting Phase Averaged wave calculated from the 10-wave window.

The raw velocity data were preprocessed using the acceleration thresholding method^47^. As current models focus on the dominant velocity component, i.e. the horizontal component *u*, this study thenceforth focuses solely on this component. To obtain a clear and homogeneous minimum and maximum value of the oscillating horizontal orbital velocity, a fourth-order zero-phase digital filter was utilized to remove any residual spikes in the data. Phase-averaging was used to find the maxima and minima of wave velocities ($U_w^{\textrm{max}}$, $U_w^{\textrm{min}}$) for each wave run. Fig. 2 shows an example of data processing for one ADV within one wave run. The resulting phase-averaged wave was then used to calculate the spatial phase-averaged steady current $U_c$ and the root mean square wave velocity $U_w^{\textrm{rms}}$ utilizing Eqs. (13) and (14) (Table 2), respectively, with $\varphi$ the phase^31^.

13 $$\begin{aligned}{} & {} U_c=\frac{1}{2\pi }\int _{0}^{2\pi } U_i(\varphi )d\varphi \end{aligned}$$

14 $$\begin{aligned}{} & {} U_w^{\textrm{rms}}=\sqrt{\frac{1}{2\pi }\int _{0}^{2\pi }(U_i(\varphi )-U_c)^2d\varphi } \end{aligned}$$

Note that in Eq. (14), $U_{w}^{{{\text{rms}}}}$ represents the phase-averaged root mean square wave velocity, whereby recently, other authors have opted for the calculation of the wave velocity amplitude, obtained by multiplying the arguments within the square root by 2^40,48^. Throughout this paper, the wave velocity amplitude refers to the minimum and maximum of the measured phase averaged wave velocity ($U_w^{\textrm{min}}$ and $U_w^{\textrm{max}}$, respectively). $U_{{\infty ,w}}^{{{\text{rms}}}}$ was taken from the measurements at position 1 (leading edge of the meadow) utilizing the fitted velocity following Eq. (15), with $b_1$ and $b_2$ as fit coefficients^49^.

15 $$U_{{\infty ,w}}^{{{\text{rms}}}} (z) = b_{1} \cdot \cosh b_{2} z$$

To obtain comparable in-canopy root mean square velocities, the fitted profiles were integrated along the canopy layer:

16a $$\hat{U}_{w}^{{{\text{rms}}}} = \frac{1}{{h_{c} }}\int_{0}^{{h_{c} }} {U_{w}^{{{\text{rms}}}} } dz$$

16b $$\hat{U}_{{\infty ,w}}^{{{\text{rms}}}} = \frac{1}{{h_{c} }}\int_{0}^{{h_{c} }} {U_{{\infty ,w}}^{{{\text{rms}}}} } dz$$

Canopy flow attenuation $\alpha _w$ was then calculated utilizing Eq. (12). Predicted values of $\alpha _w$ were obtained by solving Eq. (10), with Eq. (11) used to calculate $A_\infty ^{\textrm{rms}}$. The friction coefficient $C_f$ was previously calculated during the analysis of anchor forces^20^, with a general formulation as a function of $U_w$ obtained as a result (Eq. (17)). Note that previous studies have used a constant $C_f$ of $O(-2)$ (commonly 0.01^49^). Finally, $C_M$ was obtained following $C_M=1+k_m$, where $k_m$ is the added mass and can be estimated as $k_m=b_v/t_v$ for rectangular cross-sectional shapes^49^. This resulted in a value of 4.53 for our experiments.

17 $$\begin{aligned} C_f=0.369e^{-72.6U_w^{\textrm{max}}}+0.063e^{-3.3U_w^{\textrm{max}}} \end{aligned}$$

For the calculation of wave decay, the measured wave height of USS1 was set as $H_0$. The ratio of the average measured wave height to $H_0$ at each further position was then calculated. Eq. (1) was fitted to the calculated $H/H_0$ ratios as a function of *x* for cases with one and two ASG mats separately. $\beta$ was then obtained from the fitted curve.

For comparison with existing models, *Re* was calculated through Eq. (4) using the canopy-integrated velocities (Eq. (16a)). $l_e$ was calculated based on the scaling parameter *CaL*^22^, where *Ca* is the Cauchy Number and *L* the relative velocity between blade and water (for details see Villanueva et al.^20^). $C_D$ was then calculated utilizing Eq. (3) for pure wave conditions ($n_1=0.08$, $n_2=50000$ and $n_3=2.2$)^33^. Finally, Eq. (18)^33^, a modification of Eq. (2), was used to calculate $\beta$.

18a $$\begin{aligned} \beta&=\frac{B_1H_0}{B_2} \end{aligned}$$

18b $$\begin{aligned} B_1&=\frac{2}{3\pi }\rho C_Db_vNH_0 \left( \frac{gk}{2(\omega -U_ck)}\right) ^3\frac{\sinh ^3kl_{e}+3\sinh kl_e}{(3k\cosh ^3{kd})} \end{aligned}$$

18c $$\begin{aligned} B_2&=\biggr [\frac{\rho g}{8}\left( 1+\frac{2kh}{\sinh 2kh}\right) \left( \frac{g}{k}\tanh kh\right) ^{\frac{1}{2}}+\frac{\rho g}{8}U_c\left( 3+\frac{4kh}{\sinh 2kh}\right) \nonumber \\&+\frac{3\rho k}{8}U_c^2\left( \frac{g}{k}\coth kh\right) ^{\frac{1}{2}}\biggr ]\left[ U_c+\frac{1}{2}\left( 1+\frac{2kh}{\sinh 2kh}\right) \left( \frac{g}{k}\tanh kh\right) ^{\frac{1}{2}}\right] \end{aligned}$$

Eq. (18a) is a versatile formulation that can be applied under wave-current conditions. $U_c$ was calculated utilizing Eq. (7) (Table 2); note, however, that the lack of an additional, externally input steady current means that $U_c$ is orders of magnitude lower than $U_w$ and represents only the wave-induced underlying current. This reduces Eq. (18a) to a form analog to Eq. (2). Nevertheless, Eq. (18) yielded higher $\beta$ values than those of Eq. (2) and were more comparable to the measured (fitted) $\beta$.

## Results

Table 2 shows the results of the measurements and the respective calculations. Measurements of WR3 ($H=0.19$ m and $T=4$ s) for both 1 and 2-mat configurations presented results that clearly indicated errors in measurements and were therefore omitted from the results presented below. The wave conditions chosen fell within the intermediate water regime, close to the transition to shallow water regime following $0.003\le d/gT^2\le 0.08$.

**Table 2:** Calculated Parameters. $H_0$ represents the incident wave height, i.e. at the leading edge of the meadow (position 1, Figure 1). Non-zero numeric subscripts of *H* and *U* indicate the device position. WR3 was omitted due to anomalies in the measurements.

	WR	$H_0$	$H_2/H_0$	$H_4/H_0$	$U_{c,1}$	$\hat{U}_{\infty ,w}^{\textrm{rms}}$	$\hat{U}_{w,2}^{\textrm{rms}}$	$l_e/h_c$	$A_{\infty }^{\textrm{rms}}$	$\alpha _w$	$C_D$	$C_f$	*Re*	*KC*	$\beta$
		[m]	[-]	[-]	[m s$^{-1}$]	[m s$^{-1}$]	[m s$^{-1}$]	[-]	[m]	[-]	[-]	[-]	[-]	[-]	[m$^{-1}$]
1 Mat	1	0.09	0.96	0.96	-3.70e-04	0.10	0.10	0.50	0.03	0.97	2.87	0.09	1.27e+04	42	0.008
	2	0.05	1.27	0.91	1.77e-05	0.07	0.05	0.61	0.03	0.79	2.87	0.08	1.04e+04	42	-0.005
	4	0.11	0.12	1.22	-1.80e-04	0.15	0.11	0.59	0.12	0.76	1.95	0.10	2.17e+04	154	0.060
	5	0.18	0.98	0.92	4.45e-05	0.21	0.19	0.44	0.07	0.91	2.26	0.12	2.28e+04	86	0.006
	6	0.11	0.85	0.86	1.14e-04	0.10	0.12	0.54	0.05	1.15	2.49	0.09	1.40e+04	65	0.013
	7	0.07	0.72	0.91	-8.73e-05	0.06	0.07	0.71	0.04	1.27	2.77	0.08	9.96e+03	47	0.034
	8	0.20	0.93	1.14	4.29e-04	0.25	0.25	0.52	0.20	1.00	1.95	0.14	3.20e+04	257	0.035
	9	0.29	1.00	0.90	9.63e-04	0.24	0.20	0.44	0.08	0.84	2.15	0.14	2.69e+04	101	0.007
	10	0.23	0.94	0.87	2.43e-04	0.19	0.18	0.47	0.09	0.93	2.03	0.12	2.26e+04	119	0.006
	11	0.11	0.93	1.06	-1.47e-04	0.10	0.09	0.57	0.06	0.93	2.29	0.09	1.43e+04	83	0.006
	12	0.05	0.90	0.97	7.60e-05	0.05	0.05	0.76	0.04	0.99	2.62	0.08	1.01e+04	55	0.008
2 Mats	1	0.09	0.94	0.88	2.16e-05	0.10	0.10	0.52	0.03	0.96	2.85	0.09	1.35e+04	43	0.020
	2	0.04	1.34	0.79	7.64e-05	0.09	0.06	0.59	0.04	0.68	2.61	0.09	1.32e+04	56	-0.012
	4	0.12	0.09	1.08	2.60e-05	0.13	0.11	0.57	0.11	0.84	1.95	0.10	1.93e+04	140	0.040
	5	0.19	0.98	0.91	2.29e-04	0.20	0.18	0.45	0.06	0.90	2.29	0.12	2.27e+04	83	0.010
	6	0.10	0.96	0.93	5.24e-05	0.11	0.11	0.53	0.05	1.02	2.44	0.09	1.44e+04	68	0.012
	7	0.07	0.72	0.74	-1.40e-05	0.05	0.08	0.69	0.03	1.45	2.80	0.08	9.35e+03	45	0.045
	8	0.20	0.87	0.11	1.60e-04	0.25	0.25	0.53	0.20	1.00	1.95	0.14	3.25e+04	255	0.060
	9	0.29	1.00	0.89	1.05e-03	0.24	0.21	0.45	0.08	0.85	2.15	0.14	2.72e+04	100	0.007
	10	0.20	1.10	1.17	3.96e-04	0.20	0.16	0.47	0.10	0.80	1.99	0.12	2.40e+04	128	-0.012
	11	0.12	0.66	0.80	1.02e-04	0.08	0.12	0.60	0.05	1.57	2.50	0.08	1.16e+04	64	0.045
	12	0.06	0.53	1.07	1.06e-04	0.04	0.07	0.81	0.03	1.66	2.85	0.09	8.37e+03	43	0.016

Bed interaction and flume dimensions under the chosen wave conditions resulted in vertically asymmetric waves, most of which fell under the category of Stoke’s second and third-order theory, and those with $T\ge 3$ s and $H>0.1$ m transitioning to cnoidal waves. This asymmetry could be observed in $\eta (t)$ and $U_w$. The measured *H* thus represents the sum of the maxima and minima of the phase-averaged wave. For the orbital velocities, the rms-velocity $U_{w}^{\textrm{rms}}$ was used to obtain the results described below unless otherwise specified.

### Wave decay

Wave evolution for the control experiments (i.e. in the absence of ASG) showed that ramp-induced shoaling increased the wave height at USS1 relative to USS6 by around 3%. Viscous dissipation caused by the walls and bed could also be observed through wave decay within the control experiments. The average rate of wave decay between positions 1 and 5 for all wave runs of the control experiments was 1.9±1.5% m$^{-1}$.

The incident wave heights from all measurements with ASG ranged from 5–31 cm in front of the sand bed (USS6) and 4.5–29 cm at the leading edge of the first meadow (USS1). The ratio $H/H_0$ was close to unity at the positions in front of the sand bed, with a mean of 0.95±0.23 for all runs with ASG, while at positions above the sand bed, this lowered to 0.84–0.9±0.22 (positions 2–5). The rate of wave decay above the sand bed was 2.6±1.9% m$^{-1}$ under the presence of a single ASG mat, and 3.1±2.4% m$^{-1}$ when both ASG mats were present. The decay rate was highest between positions 1 and 2, at 5.6 and 7.2% m$^{-1}$ for 1 and 2 mats, respectively, suggesting that the first mat relative to the wave propagation direction has a more marked effect on wave decay.

**Figure 3:**
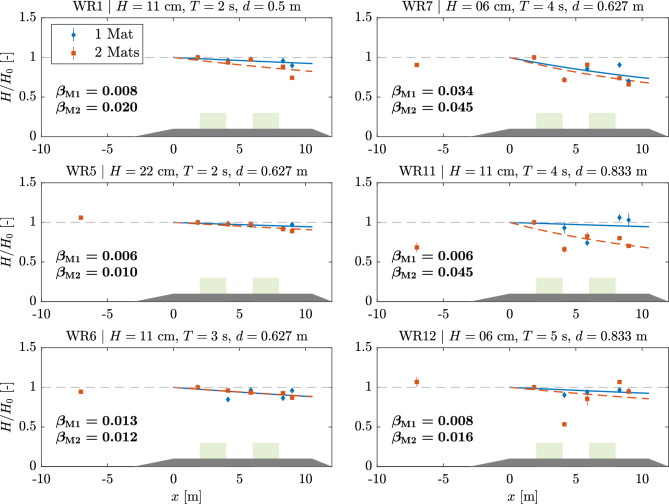
Wave Decay evolution for different wave runs. Data points show the average and standard deviation of $H/H_0$ for each USS over the sand bed and at position 6 in front of the sand bed. $H_0$ is taken from USS1 at the leading edge of the meadow. Fits follow Eq. (1) with solid line representing runs with one mat (subscript M1) and dashed line runs with two mats (subscript M2). The corresponding resulting $\beta$ is given for each run. Shaded areas show ramp and ASG meadows (not to scale).

Fig. 3 shows the average wave height ratio evolution ($H/H_0$) along *x* for 6 different runs segregating between one and two-mat configurations. The average of ratios for all runs with ASG is presented to encompass all measurements. The damping coefficient $\beta$ was calculated by fitting Eq. (1) to the wave height data, resulting in values ranging between -0.005–0.06 m$^{-1}$ and -0.012–0.06 m$^{-1}$ for 1 and 2-mat configurations, respectively (Table 2). The set of 1-mat experiments averaged $\beta =0.0161\pm 0.018$ m$^{-1}$, whereas for the 2-mat experiments $\beta =0.021\pm 0.023$ m$^{-1}$. A second mat then enhanced wave decay, with $\beta$ for the 2-mat configuration being on average 30% higher than for the 1-mat configuration.

Although $\beta$ shows that wave decay is augmented by the mats, the variability of the values (Table 2) indicates that the input parameters have a meaningful effect on wave decay. As shoot and base layer morphology were not modified during the experiments, the incident wave conditions represent the governing variables. Analysis of the relationship between $\beta$ and the incident wave height $H_0$ showed no correlation for the wave conditions tested here. A simple linear regression between the water depth *d* and $\beta$ also showed no significant correlation between both variables ($\textrm{p}>0.05$). However, a light tendency of $\beta$ decreasing with increasing *d* was observable, showing a low rate of change of -0.0048 per 10 cm of added water depth for the 1-mat experiments and -0.0019 dm$^{-1}$ for 2 mats. Regardless, the low rate and correlation indicate that the submergence ratios trialed here, i.e. $h_c/d=[0.3,0.4,0.5]$, had little effect on wave attenuation for the wave conditions trialed.

**Figure 4:**
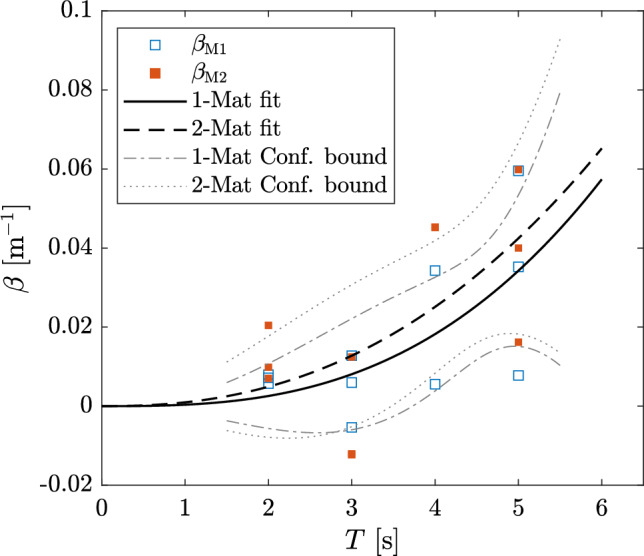
Damping coefficient $\beta$ plotted against the incident wave period *T*. Values are shown for 1 and 2 ASG mats (M1 and M2, respectively). Lines show fit for each configuration (Eq. (19)) and the corresponding confidence bounds.

In contrast, analysis of the relation between the wave period *T* and $\beta$ showed that $\beta$ increased exponentially with increasing *T*. The relationship between $\beta$ and *T* makes it obvious that $\beta$ is analogously sensitive to the wavelength $\lambda$. Fig. 4 shows the change of $\beta$ with respect to *T* for both mat configurations. An exponential fit for each set of experiments was done to showcase the effect of *T* on wave decay, with the respective fits given in Eq. set 19.

19a $$\begin{aligned} \beta _{M1}&=0.000361T^{2.83} \vert R^2=0.44 \end{aligned}$$

19b $$\begin{aligned} \beta _{M2}&=0.000963T^{2.35} \vert R^2=0.41 \end{aligned}$$

Note, however, that the variance of the fitted $\beta$ with respect to *T* is still relatively high, with $R^2<0.5$ for both cases. The limited data and variability of the calculated $\beta$ produce fits with a high prediction variability within the confidence intervals, as shown in Fig. 4. Therefore, it is important to keep in mind that although Eqs. (19a,b) may provide insight into an increasing damping coefficient with respect to *T*, this is explicitly valid for wave decay above meadows of similar geometric and mechanical characteristics and wave conditions to those trialed here, i.e. within the range $1<T<6$ s.

#### Modeled damping coefficient

The calculation of $\beta$ requires an estimation of the drag coefficient $C_D$, which, for vegetated flow, has been commonly related to the Reynolds Number *Re* and Keulegan-Carpenter Number *KC*. The range of canopy-integrated rms-velocities $\hat{U}_w^{\textrm{rms}}$ measured here yielded values of $Re^{l_e}$ between 8300–32500 and *KC* between 42–260. The ratio of effective length to upright canopy height ($l_e/h_c$) ranged between 0.44–0.81. Values of the stem-based *Re* (i.e. based on $b_v$) were *O*(1) lower, ranging between 200–1200. The calculation of $C_D$ following Eq. (5) yielded values between 1.95–2.96 (mean $=2.4$).

**Figure 5:**
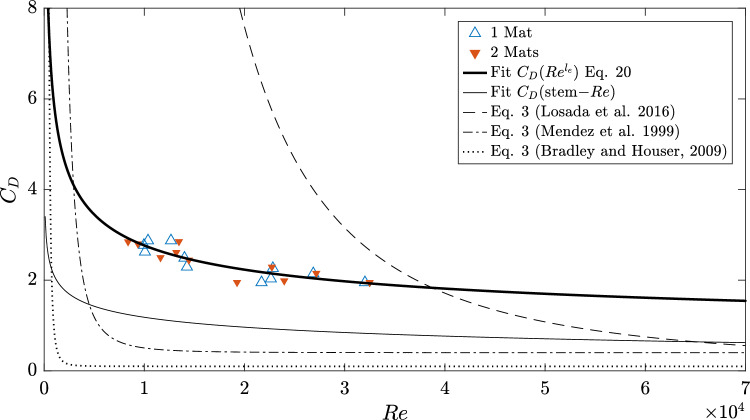
Drag coefficients (Eq. (5)) as function of $Re^{l_e}$ (Eq. (4)) and resulting fit (solid black line, Eq. (20)). Dotted line shows the fit for $C_D$ as a function of the stem-*Re*. Results of Eq. (3) using the coefficients: [$n_1=0.08, n_2=50000,$ and $n_3=2.2$]^33^, [$n_1=0.40, n_2=4600,$ and $n_3=2.9$]^50^, and [$n_1=0.1, n_2=925,$ and $n_3=3.16$]^23^ are given for comparison.

Fig. 5 shows $C_D$ plotted as a function of $Re^{l_e}$. Results from other studies with different experimentally fitted $C_D=f(Re)$ following Eq. (3) are shown for comparison. For the present set of experiments, the corresponding fit is shown resulting in the coefficients shown in Eq. (20) ($R^2=0.79$). The different results show how sensitive $C_D$ is to the choice of characteristic length and input velocity, directly reflected in the variation of the scale of *Re*.

20 $$\begin{aligned} C_D=0.31+\left( \frac{127000}{Re^{l_e}}\right) ^{0.35} \end{aligned}$$

**Figure 6:**
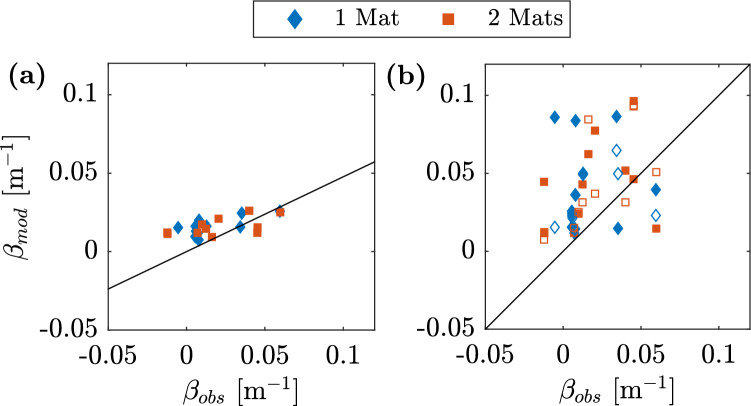
Predicted (subscript *mod*) versus measured (subscript *obs*) wave damping coefficient $\beta$. (a) $\beta$ modeled after Eq. (2). Solid line represents a linear fit with intercept at (0,0). (b) comparison with results from Eq. (18)^33^. Solid line represents 1:1 line. Hollow markers represent the results from the model of Lei and Nepf^32^ for comparison.

The modeled damping coefficient $\beta _{mod}$ was then calculated based on $C_D$ (Eq. (5)) following Eq. (2). Fig. 6 shows the comparison between the modeled values and the values obtained from measurements ($\beta _{obs}$). A linear fit between modeled values and measurements showed that the model underestimates the damping coefficient by 52% (based on the slope of the linear fit, no intercept). A comparison with modified forms of Eq. (2) is also shown in Fig. 6: Eq. (18)^33^ overestimated $\beta _{obs}$, with $\beta _{mod}=1.15(\pm 0.32)\beta _{obs}$, while a further modification^32^ (discussed later) yielded $\beta _{mod}=1.30(\pm 0.21)\beta _{obs}$. Both models underestimated $\beta _{obs}$ for wave runs with the longest periods, i.e. $T=5$ s, and wave heights above 0.1 m. A similar pattern was observable with the model after the original formulation (Eq. (2)), where a 1:1 comparison between modeled and measured values showed that the $\beta _{mod}$ underpredicts $\beta _{obs}$ for all but one of the wave runs with $T\ge 4$ s.

### Velocity structure

The maximum measured orbital velocities $U_w^{\textrm{max}}$ increased proportionally with the increase of *H*, as is expected from wave theory, with no correlation to either *T* or *d* found for the range of conditions trialed. Comparison of $U_w^{\textrm{max}}$ with the maximum velocities measured in the opposite direction (i.e. $U_w^{\textrm{min}}$) showed that the non-zero net momentum transport in the direction of wave propagation (i.e. Stoke’s Drift) was present regardless of the number of mats (0, 1 or 2) and ADV position (1–4). ASG thence did not alter the overall mass transport in the direction of wave propagation, demonstrated by the ratio $U_w^{\textrm{max}}/U_w^{\textrm{min}}$, which, averaging for each set of measurements, yielded $1.34\pm 0.22$ for the control experiments, and $1.36\pm 0.19$ and $1.37\pm 0.23$ for the one and two-mat configurations, respectively. Note that Stoke’s Drift may be an artefact of the wave flume; however, its analysis is out of the scope of this study and is therefore not discussed further.

**Figure 7:**
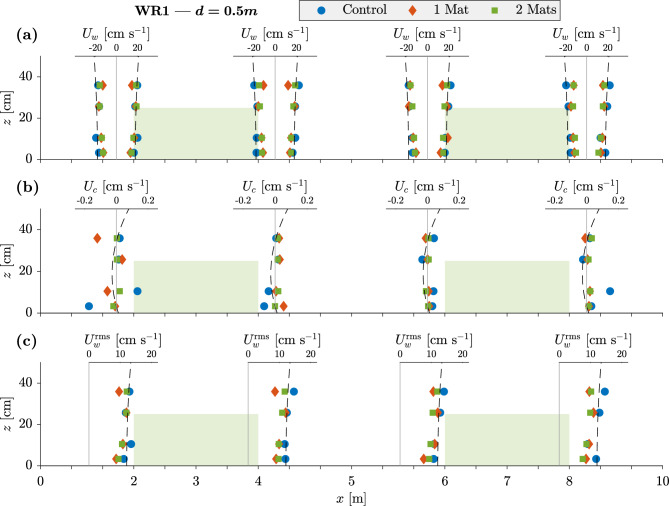
Velocity Structure for WR1 ($H=0.11$ m, $T=2$ s). Profiles show *x*-*z* position of ADV measurements. Data points show measured values for control (no ASG mats), 1-mat and 2-mat experiments. (a) fully measured wave velocity excursion showing maxima ($U_w^{\textrm{max}}$) and minima ($U_w^{\textrm{min}}$); dashed fitted line shows theoretical profile following Eq. (15). (b) Steady current component of flow $U_c$ calculated from measurements using Eq. (13); dashed line represents Eq. (7). (c) Root mean square velocity calculated from measurements and phase-averaged using Eq. (14); fitted line from Eq. (15). Shaded areas show ASG at full height $h_c$.

**Figure 8:**
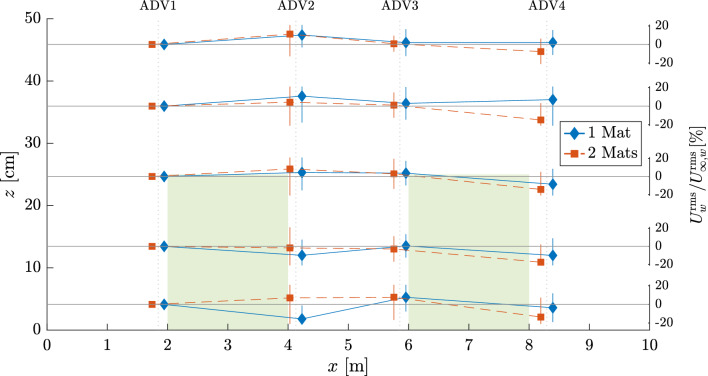
Percentage change in $U_w^{\textrm{rms}}$ with respect to measurements at the leading edge of the meadow (position 1). The average percentage change of all wave runs is shown. Vertical bars at each point represent standard deviation. Markers deviate slightly from ADV position axes for clarity. Shaded areas show ASG at full height $h_c$. Note that the lines joining each *x*-marker only qualitatively represent the trend between the positions, as changes in $U_w^{\textrm{rms}}(z)$ are not actually linear and would require more information within each meadow to depict their true behavior.

Fig. 7a shows the range of $U_{w}$ at each ADV position for WR1 ($H=0.11$ m, $T=2$ s), whereby the aforementioned asymmetry becomes greater for higher *T* as non-linearity effects intensify. The canopy-integrated velocities ranged from 0.35–0.53 m s$^{-1}$ for M0, 0.34–0.48 m s$^{-1}$ for M1, and 0.33–0.46 m s$^{-1}$ for M2. The highest velocities in the direction of wave propagation were measured exiting the meadows, i.e. at positions 2 and 4, while in the opposite direction, the highest velocities were at position 1, i.e. exiting the first meadow at the leading edge. Overall, WR8 ($H=0.22$ m, $T=5$ s, and $d=0.63$ m) recorded the highest velocities. Fig. 7b shows $U_c(z)$ (Eq. (13)), whose magnitude was predominantly within the range of $O(-4)$ and $O(-5)$. The highest values were observed for M0 ($O(-3)$), though the near-bed velocities of both ASG experiments likewise reached $O(-3)$. At the leading edge of the meadow, a peak in the positive direction could be observed within $z\le 10$ cm for WR2, 5, 9, and 10. The theoretical model^41^ (Eq. (7)) predicted values in the same order of magnitude of the measurements for the lowest energy runs (i.e. $H<0.1$ m). However, neither the shape of the profile nor the highest magnitudes were captured well by the model which overestimated $U_c(z)$ for $H>0.2$ m by up to *O*(1). Fig. 7c shows the profiles of $U_w^{\textrm{rms}}(z)$. The dashed line was fitted as a function of *z* at position 1 (Eq. (15)) and is thence shown at each further *x*-position for reference. $U_w^{\textrm{rms}}(z)$ decreased with increasing distance along *x*, with changes along *x* becoming more conspicuous as the velocities increased. To better visualize the effect of the meadows on $U_w^{\textrm{rms}}$, the percentage change in velocity relative to position 1 was calculated and is shown in Fig. 8. The velocity profiles show that the vegetation induces skimming flow, which is the result of discontinuity in the drag force and mass balance along the meadow. Skimming flow has been shown for wind profiles around urban canopies^51^ and submerged vegetation as a function of wave conditions^52^.

Analogous to $H/H_0$ (Fig. 3), Fig. 8 shows the evolution of $U_w^{\textrm{rms}}(z)$ along *x* with respect to position 1 (i.e. $U_{\infty ,w}^{\textrm{rms}}$), calculated as: $[(U_w^{\textrm{rms}}(x,z)/U_{\infty ,w}^{\textrm{rms}}(x,z))-1]*100$. Negative values represent velocity reduction, while positive values indicate an increase. The values are averaged from all wave runs, with deviations depicted by vertical bars. The deviation ranged between 8–20%, except at position 2 for M2, where the deviation reached on average 30% at all $z_i$. Deviations $>20\%$ were also measured at $z_4$ at all *x*-positions for M1, and just over 20% at $z_1$ for M2 at positions 3 and 4. The reduction of $U_w^{\textrm{rms}}$ along *x* ranged from 2–17.5% and was more prominent directly behind the ASG mats. Increases in $U_w^{\textrm{rms}}$ ranged from 0.5–11%, with the highest values ($\ge 10\%$) in the region above the meadow ($z_4, z_5$), which indicates an increase in velocity resulting from the presence of ASG. For 2 mats, there was an increase (7–7.7%) in the near-bed velocities within the gap, whereas with 1 mat, the same value (7%) was observed at position 3, but rather increasing from a reduction ($-15.6\%$) at position 2. With increasing distance behind the first mat, $U_w^{\textrm{rms}}$ decreased again, regardless of mat configuration. However, the second mat enhanced this reduction, which was additionally accentuated by the proximity to the bed, with M2 reducing 10, 7.5, and 5.7% more than M1 for $z_1$, $z_2$, and $z_3$, respectively. This tendency to increase after the first mat and gradually reduce as waves go through the second mat was observed along the full transect for M2. For M1, the velocities sink directly behind the ASG mat and subsequently set up immediately afterward, a behavior that is only prominently observed within the canopy height. Above the canopy, the velocities for M1 remain on average relatively stable.

**Figure 9:**
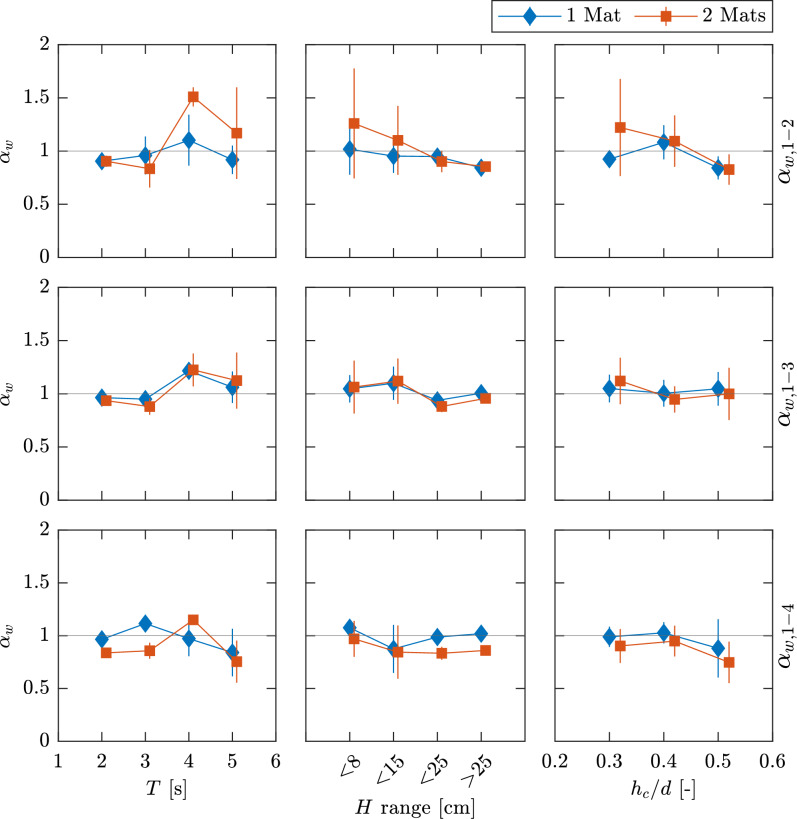
Variation of the flow attenuation parameter $\alpha _w$ depending on the hydrodynamic parameters and the position of measurement: wave height *H*, wave period *T*, and water depth *d* shown in columns 1, 2, and 3, respectively. Position of $\alpha _w$ shown by rows (subscripts on the right indicate position). Deviation of measurement given by vertical bars. *H* is shown for a range of measured wave heights.

The flow attenuation parameter $\alpha _w$ is analogous to the percentage change shown in Fig. 8, depicting the evolution of $\hat{U}_w^{\textrm{rms}}$ (Eq. (16)). $\hat{U}_w^{\textrm{rms}}$ ranged from 0.04–0.29 m s$^{-1}$ for M0, and from 0.04–0.25 m s$^{-1}$ for M1 and M2. The maximum value was consistent at all ADV positions for M1, but lowered to 0.22 m s$^{-1}$ for M2 at position 4, showing a reduction of $\hat{U}_w^{\textrm{rms}}$ along *x*. Table 2 shows $\alpha _w$ calculated between positions 1 and 2, however, like $U_w^{\textrm{rms}}(x,z)$, $\alpha _w$ displayed variation along *x*. Increases in $\hat{U}_w^{\textrm{rms}}(x)$ were recorded, leading to $\alpha _w>1$, thus indicating flow enhancement instead of attenuation. The variability exhibited by the spread of the velocity data (Fig. 8) and $\alpha _w$ indicates that the incident wave conditions greatly alter the velocity structure (as vegetation parameters did not vary). To assess the effect of the incident hydrodynamics, $\alpha _w$ was calculated at each *x*-position with respect to $\hat{U}_{\infty ,w}^{\textrm{rms}}$, subsequently plotting against the different instances of *H*, *T*, and *d* separately (Fig. 9). Within the uncertainties, the calculated values of $\alpha _w(x)$ generally did not show a clear trend toward gain or loss depending on *H*, *T*, or *d*. An exception can be observed for the averaged values with the addition of a second mat, especially between positions 1 and 2; however, uncertainties increased by up to 40%. For the averages, it can be seen that $\alpha _w$ decreases with increasing *H*, going from 1.26$\pm 0.52$ to 0.90±0.1 for waves >15 cm, indicating that flow is more readily attenuated for higher waves (i.e. higher velocities). Similar to $\beta$, $\alpha _w$ increased from 0.83$\pm 0.14$ for $h_c/d=0.5$ to 1.22$\pm 0.46$ for $h_c/d=0.3$ (2 mats), denoting less attenuation for increased depths (i.e. decreased submergence ratio). On the other hand, attenuation decreased, i.e. higher $\alpha _w$, as *T* increased, going from 0.90$\pm 0.05$ for $T=2$ s to 1.17$\pm 0.43$ for $T=5$ s. These tendencies were reflected less as the distance along *x* increased, with the differences between 1 and 2 mats becoming less obvious.

#### Modeled flow attenuation

**Figure 10:**
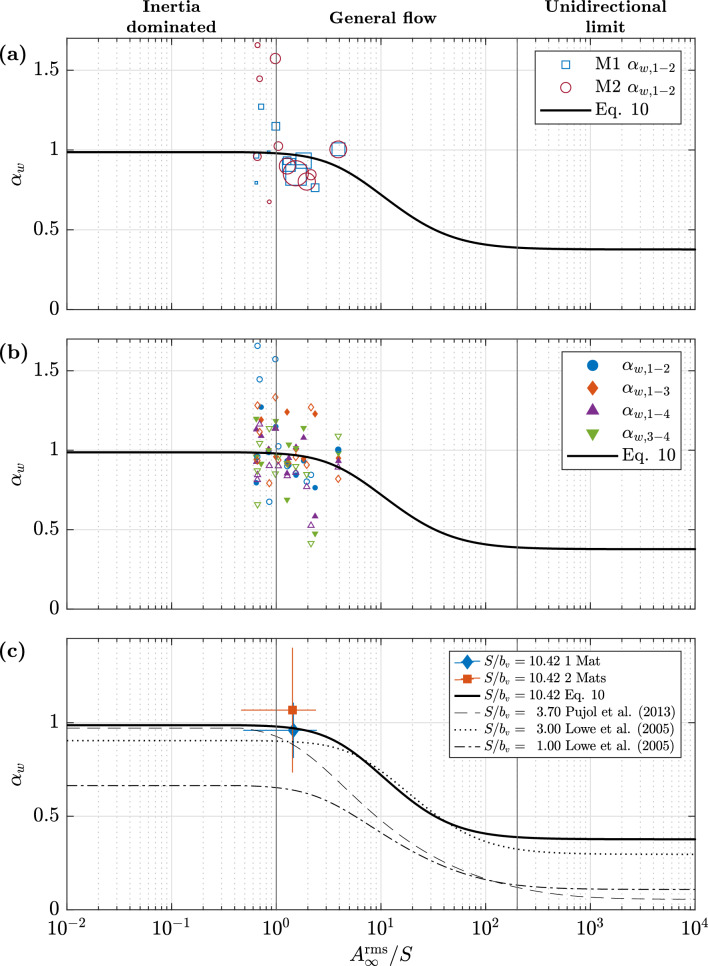
Canopy flow attenuation parameter $\alpha _w$ as a function of the ratio of wave orbital excursion and stem separation $A_\infty ^{\textrm{rms}}/S$. Solid line represents solution to Eq. (10). (a) $\alpha _w$ between positions 1 and 2. Size of Markers qualitatively shows the range of wave heights measured. (b) $\alpha _w$ at all positions (denoted by subscripts). Hollow markers represent 2-mat experiments. (c) Comparison of the solution to Eq. (10) for different studies with varying plant characteristics. Mean and error of $\alpha _{w,1-2}$ shown for reference.

Fig. 10 shows the empirical values of $\alpha _w$ plotted against the ratio $A_\infty ^{\textrm{rms}}/S$ along with the solution to the model in Eq. (10). The wave conditions and vegetation characteristics trialed here led to a range of $A_\infty ^{\textrm{rms}}$ from 3–22 cm for M0 and 3.2–20 cm for M1 and M2. With a constant shoot separation $S=5$ cm, this resulted in the range of $A_\infty ^{\textrm{rms}}/S$ of roughly 0.6–4. This range puts the conditions trialed here at the interface between the general flow and inertia-dominated regimes^21^. The wide spread of $\alpha _w$ presented above makes it obvious that the model does not agree with a wide range of the conditions trialed here. Fig. 10a shows $\alpha _w$ between positions 1 and 2 distinguished by the number of mats and the measured wave height. It can be seen that the model generally performs better for greater wave heights ($H>0.15$ m), with lower wave heights both over and underpredicting $\alpha _w$. As before, this was accentuated for M2.

Fig. 10b shows $\alpha _w$ at all positions. The differentiation between 1 and 2 mats reveals that over and underprediction by the model is more likely to occur under the presence of a second mat, with only 36% of the M2 runs falling within ±10% of the modeled $\alpha _w$ compared to 55% for M1. Fig. 10c shows the solution to Eq. (10) from studies with different input hydrodynamic conditions and vegetation parameters for comparison with the present set of experiments. For reference, the average value of the set of wave runs for M1 and M2 are shown, where it can be observed that although the set of experiments agrees well with the model for the average (especially for 1 mat), the uncertainty is high for varying hydrodynamic conditions.

## Discussion

### Wave dynamics around flexible ASG mats

The velocity profiles showed the presence of a wave-induced current whose profile $U_c(z)$ was modified by the flexible ASG mats. For rigid surrogates, it has been shown how $U_c(z)$ shifts direction at canopy interfaces (i.e. canopy top and meadow edges)^53^, while a return current, essential for nutrient circulation within the meadow, has been observed for flexible vegetation^31^. Here, these behaviors were only observed at certain interfaces, yet not as a common occurrence. Nonetheless, like previous studies^49^, the profiles of $U_c(z)$ around flexible vegetation are of similar form to non-vegetated conditions. This is because *N* was too low to properly modify the wave-induced current, which easily penetrated into the canopy.

The control experiments showed that the measured wave decay encompassed energy loss not only due to vegetation but also static continuous losses, i.e. losses due to wall and bed friction. The choice of $H_0$ in Eq. (1) is thus not inconsequential. Further, previous studies^29,31^ emphasize the assumption that energy dissipation and the ensuing wave decay stem mostly from drag-induced forces caused by vegetation, whereby the current paradigm sets $H_0$ as the incident *H* (leading edge of meadow). Hence, neither the non-vegetated wave height nor the effect of continuous losses along *x* is taken into account. For comparison, $\beta$ calculated based on *H* of the control experiments (M0) at each position (effectively removing continuous losses) ranged from 0–0.03 m$^{-1}$, or half the range presented above ($-0.01<\beta <0.06$ m$^{-1}$) . Moreover, if we take $H_0$ as the incident *H* of M0, $\beta$ ranges from -0.01–0.13, demonstrating an additional effect of the ASG compared to non-vegetated areas. These differences are particularly important when intending to apply empirical models developed under laboratory conditions to field applications.

With a low shoot density, a flexible base layer, and single-stem flexible shoots, the measurements indicate that a sparse, fully flexible canopy is able to dissipate energy. This in turn could promote seagrass growth^17^. The degree of attenuation is clearly dependent on vegetation properties and incident hydrodynamic conditions and can vary greatly between set-ups. For example, Losada et al.^33^ obtained a range of $0.02\le \beta \le 0.32$ m$^{-1}$ (*O*(1) higher than this study) for experiments with circular patches of *S. anglica* and *P. maritima* of varying rigidity, whereby, compared to this study, density was up to 3 times higher, $h_c/d\ge 0.5$ (including emergent conditions), and $1.7\le T\le 2.2$ s (see Supplementary Table S1). Here, $\beta <0$ for 3 wave runs (all with $T=3$ s, Table 2), which result from $H/H_0>1$ at certain positions. Flow attenuation displayed a similar behavior ($\alpha _w>1$), indicating increased flow around the meadows. The wave setup and enhanced flow were certainly initiated by the obstructing structure, i.e. ASG, but could be boosted by mat motion. Furthermore, the interaction between the oscillatory flow and the meadow at position 1 could lead to an increase of $\hat{U}_{w}^{\textrm{rms}}$ with respect to $\hat{U}_{\infty ,w}^{\textrm{rms}}$. Of course, this has not been observed as a common phenomenon in other experimental studies, which suggests that the increased mat flexibility and added mat motion may be responsible for the reduced attenuation.

Moreover, with measurements taken at canopy interfaces, the measured $U_w$ is affected by a vegetated side and a non-vegetated side. The effect of the bare side is particularly evident within the gap hydrodynamics, where, within the canopy height, in-gap measured velocities varied depending on whether a second mat was present further downstream. The evolution of $U_w^{\textrm{rms}}/U_{\infty ,w}^{\textrm{rms}}$ shown in Fig. 8 shows how, with one mat, the attenuation of flow was enhanced directly behind the mat, and velocities increase rapidly before steering toward a steadily attenuated flow downstream. With 2 mats, however, the first mat does not have the same attenuating effect per meter, but rather present a shift of the attenuated velocities toward the end of the second meadow. This suggests that the 2 mats behave as a single meadow, with the highest attenuation at the trailing edge. Furthermore, skimming flow becomes apparent at the canopy interfaces, gradually attenuating flow further down the wave propagation direction^54^. Savio et al.^55^ showed for aligned patches that skimming flow develops roughly at gap width equal to patch length, consistent with the measurements here. On the other hand, a similar study on gap hydrodynamics^56^ found that wave velocities were reduced after the first meadow and returned to pre-vegetated conditions for gaps longer than $2h_c$. This was not corroborated here (gap length: $8h_c$) as $U_w^{\textrm{rms}}$ showed no attenuation within the gap, but further down the meadows.

**Figure 11:**
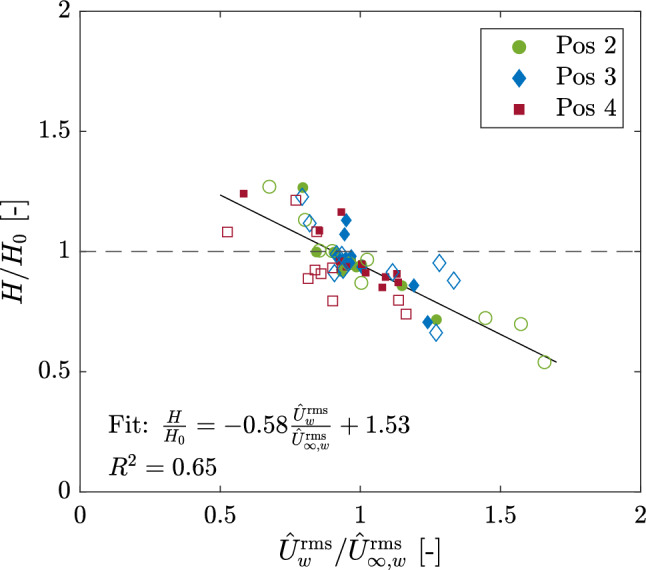
Relation between wave evolution $H/H_0$ and flow attenuation $\hat{U}_w^{\textrm{rms}}/\hat{U}_{\infty ,w}^{\textrm{rms}}$ at all positions (Pos) relative to position 1. Hollow markers indicate 2-mat configuration. Solid line represents linear fit.

Perhaps an interesting outcome to discuss with regard to wave decay and flow attenuation is the relationship between both. Fig. 11 shows the relation between $H/H_0$ and $\hat{U}_w^{\textrm{rms}}/\hat{U}_{\infty ,w}^{\textrm{rms}}$ for the complete set of experiments (all positions). A linear fit was done for the set of 66 points (4 outliers removed due to unlikelihood). Fig. 11 shows that measurements with higher wave attenuation (i.e. lower $H/H_0$) yielded lower flow attenuation (higher $\alpha _w$), while higher flow attenuation resulted in lower wave attenuation. In general terms, this suggests that as velocities around the meadows are more actively reduced, wave heights are less reduced with respect to the incidence. This is consistent with Lowe et al.^57^, who found that the rate of wave dissipation increases with increasing flow through the meadow, i.e. higher $\alpha _w$. Therefore, as flow penetrates more readily into the meadow, wave heights are more actively reduced.

#### Effect of incident hydrodynamic conditions

Studies have shown that wave energy dissipation can vary depending on the wave period^38^, higher submergence ratios $h_c/d$ lead to higher wave attenuation^33,54^, and vegetation geometric characteristics (e.g. $\lambda _f, \lambda _p$) play an essential role^58^. Here, other than the variation of $h_c/d$, the constant vegetation traits remained independent of the fluctuating hydrodynamics. The high variability exhibited by the results thus indicates that the hydrodynamic conditions greatly affect wave and flow attenuation. Previous studies have shown that the rate of energy dissipation is highly dependent on *T*^10,23,57^ , with shorter wave periods yielding higher wave attenuation and longer periods higher in-canopy flow attenuation. The relationship between $H/H_0$ and $\alpha _w$ shown in Fig. 11 corroborates this inverse behavior. However, as shown in Fig. 9, for $T\ge 3$ s and $h_c/d\le 0.4$, $\alpha _w$ was enhanced within the gap of the 2-mat configuration compared to the 1-mat configuration, suggesting that flow enhancement is a result of the gap. This was also reflected with $h_c/d$, where $\alpha _w$ decreased for increasing $h_c/d$ at position 2, indicating that flow within the gap becomes proportionally lower as the meadow takes more of the water column. Moreover, connecting $h_c/d$ and *T*, the measurements indicate that flow penetrates more easily into the canopy for longer wave periods as the submergence ratio decreases. The inconsistency within the results suggests that the effect of the gap on flow varies with *T* and $h_c/d$. Lara et al.^59^ found that while turbulent diffusion was enhanced by heterogeneous meadows (i.e. with gaps), flow velocities were not enhanced by meadow patchiness. Moreover, Paul et al.^58^ argued that $\lambda _f$ and $\lambda _p$ are more important than a varying submergence ratio stemming from varying *d*, which, for $h_c/d\le 0.4$, was also observed here. For storm conditions, Möller et al.^39^ found that marshes attenuated waves regardless of the variable water depth. In-depth gap hydrodynamic studies are necessary to couple the effect of the gap with interchanging submergence ratios and varying hydrodynamic conditions.

### Predictive models of wave and flow attenuation

The experiments demonstrated that the flexible ASG mats attenuate waves and flow similarly to conventional (fixed) meadows, i.e. behavior and parameter sensibility are established comparably. However, the attenuation magnitude was generally lower than predictive models would suggest, with uncertainties increasing as a function of incident hydrodynamic conditions. As analytical models are generally validated experimentally to support the range of empirical values obtained, most models are also sensitive to input variability. Recent wave attenuation models based on the original model^28^ thus further incorporate intrinsic conditions such as measured in-canopy flow and plant motion. van Veelen et al.^60^ extended the model to account for vegetation flexibility and motion by including blade excursion during wave loading; they validated it for regular waves with small $A_\infty$ over cylindrical, flexible, and near-stiff vegetation, and performed well applied to real salt marshes of different stiffness (*P. maritima, S. anglica* and *E. athericus*). Further, following the findings of Lowe et al.^57^, Lei and Nepf^32^ extended Eq. (2) by factoring in flow attenuation, thus incorporating the effect of vegetation on in-canopy flow; they also incorporated $l_e$ to account for plant motion and consequent drag reduction (similar to^33^), obtaining a modified Eq. (2) as $\beta =(2/9\pi )C_Db_vNkH_0\alpha _w^3(J_1/J_2)$, where $J_1=9\sinh (kl_e)+\sinh (3kl_e)$ and $J_2=\sinh kd(\sinh (2kd)+2kd)$. Modified forms of Eq. (2) (^32,33^) yielded values of $\beta$ of the same order of magnitude as those observed here, while Eq. (2) largely underestimated them (Fig. 6). It is not surprising that the modified formulations improved the results; however, it is interesting to note that both approaches underestimated the damping coefficient for longer-period waves. With both models empirically validated for $T<2$ s, it becomes clear that future models should focus on longer-period waves, as such conditions also lead to higher flow attenuation^57^ and are more likely to dominate field conditions^23,61^. Note that shorter wave periods lead to more flow through the canopy, leading to $\lim _{T\rightarrow 0}\alpha _w=1$, which reduces the equation described above to Eq. (2).

The measurements yielded a wide range of $\alpha _w$, suggesting that Eq. (10) is not applicable to flexible ASG mats. However, it is important to remember that $\alpha _w$ measured here corresponds to the ratio at the edges of the meadows and not the in-canopy flow (measured through a clearing^21^). Knowing this, it is expected to have higher values of $\alpha _w$ than in-canopy measurements would yield. However, as the results show, $\alpha _w$ was both over and underestimated by the model, depending on wave run and position. This leads us back to the sensitivity with regard to input conditions and the role of the gap in patch-to-patch hydrodynamics. Even though the results suggest that the model should not be used to determine the flow attenuation expected by similar flexible mats, the resulting average from the ensemble of measurements indicates that for a general range of conditions, the model can provide proper insight into the effect of the canopy. Special care should still be taken regarding gap hydrodynamics, which was shown to be unstable.

Model results proved to be sensitive to the choice of input parameters, such as $C_D$, $C_f$, and $C_M$, the characteristic lengths (e.g. $b_v$, $h_c$, and $l_e$), and the resulting dimensionless quantities describing flow patterns (e.g. *Re* and *KC*). Supplementary Table S1 lists several studies that have investigated different parameters, proposing ways to calculate them depending on target conditions. Ultimately, it is important to know how a model was validated to determine which parameters are more relevant and what input they need for their calculation. For the present experiments, canopy-integrated rms-velocities were used as the characteristic velocity. The use of in-canopy velocities provides an estimation of $C_D$ which better represents vegetation-induced drag as this may also vary along *z*. This is especially true for higher frequency conditions beyond the shallow water regime, as the oscillatory velocity becomes more sensitive to *z* and the velocity at the canopy top may thus greatly differ from near-bed velocities. Note that rms-velocities are lower than the maximum velocity $U_w^{\textrm{max}}$, meaning that dependent parameters will be proportionally lower. However, $U_w^{\textrm{max}}$ does not dominate the range of velocities in a temporal scale within a given period (see^20^), especially as the frequency decreases. Thus, a better representation of the time-averaged *Re* or *KC* is given by the representative rms-velocity.

### Implications for field applications

The conditions trialed corresponded to a wide range of wave heights, periods, and depths to mimic the wide range of conditions that can be found in the field^61^. This yielded a high variability within the resulting attenuation coefficients (i.e. $\beta$ and $\alpha _w$) and associated parameters (e.g. $C_D$ and *Re*), making it difficult to assess the suitability of a single model to predict any specific effect that a field-deployed ASG mat may have on its surrounding hydrodynamics. The flexible nature of the discretely anchored mats provides higher freedom of movement (sway) than typically fixed meadows used for experimentation. For the tested mats under 2D wave excursion, Villanueva et al.^20^ showed how the forces on the anchors are effective in all 3 dimensions. Moreover, it is important to remember that field and laboratory conditions can differ greatly. Losses due to viscous dissipation from flume walls and reflection effects are not present in the field, whereas external factors such as organism interaction with the (artificial) vegetation and the spatiotemporal variability of the field conditions can render empirical models very limited in their application.

Nevertheless, the results showed that flexible ASG mats affect both wave evolution and the velocity structure along the water column, with predictive models yielding estimates adequate in magnitude for the average of a wide range of conditions. Insight into expected wave and flow attenuation can be obtained as long as flow-vegetation interaction is considered; i.e. plant flexibility and canopy-affected flow. Fragmented canopies, however, will affect the hydrodynamics and thus decrease model accuracy. While current models may provide helpful insight into the effect of single continuous meadows on local hydrodynamics, the effect of fragmented canopies as well as the interaction with random waves and waves plus current should be investigated to provide more accurate predictions of the effect of mats deployed in the field on local hydrodynamics. Furthermore, mat sway should be measured in future experiments to integrate its effect on the velocity structure in and around a meadow.

The attenuation of waves and oscillatory flow by the ASG mats indicates that they could help to promote seagrass growth. This is especially true for the 2-mat configuration, which showed increased attenuation and a single-meadow behavior when observing flow modulation. However, the enhanced gap hydrodynamics also suggest that shelter within the gap is reduced, hence settling of seedlings may be challenging. As measurements behind the second mat showed enhanced attenuation, this may be a phenomenon exclusive to the first gap (relative to the main wave propagation direction) for fragmented canopies with more than two patches. Moreover, as wave frequency decreases and flow tends to the uni-directional limit ($T\rightarrow \infty$, e.g. in tidal areas) shelter is provided in the wake of seagrass meadows^62^, so that the dominating wave conditions play an important role. Based on these results, we suggest that field experiments with artificial seagrass should employ mats that also allow for seagrass to grow within the ASG, not only aiming for in-gap growth based on wave shelter. Furthermore, for a wide range of conditions and single meadow interaction, results showed that the models can provide insight into the expected attenuation effects of the meadow, as long as uncertainties are also cross-examined and informed. Seagrass reestablishment efforts using ASG can thus be supported by existing flow-vegetation-interaction models by providing faithful magnitude projections of the effect of anchored mats in the field.

## Conclusions

Experiments were carried out in a large-scale flume to test the effect of discretely anchored flexible mats of artificial seagrass (ASG) on wave evolution and flow structure. Such mats are intended to be deployed in the field to promote seagrass growth, sheltering seedlings from harsh conditions where they would otherwise not be able to thrive. To help design these mats, an accurate understanding of their effect on the surrounding hydrodynamics is needed. For this purpose, 2x2-m mats were tested under varying hydrodynamic conditions. As several models of flow-vegetation interaction have been developed, the experiments aimed to test their suitability to predict the effect of the mats under varying hydrodynamic conditions. This in turn can help practitioners to locate potential sites for seagrass reestablishment and foresee the performance of the mats to this end.

The experiments showed that the fully flexible mats, i.e. flexible base layer and artificial vegetation stems, are able to attenuate waves and flow. However, it was also noted that these results are subject to the specific conditions trialed, i.e. hydrodynamics and vegetation properties, which represent the main limitation of the study. Further experimentation and field studies are encouraged on the basis of these results to expand on them. Regarding the experimental results, it was shown that attenuation of flow and waves was enhanced behind a second ASG mat. However, the presence of the second mat also induced flow enhancement within the gap between both mats, which suggests an important role of gap hydrodynamics and motivates further research. Further, it was found that less flow attenuation, i.e. more flow through the canopy, leads to increased wave height reduction. Current models of flow-structure interaction were not able to capture the effect of the mats for the whole range of conditions. Unstable results by contemporary models may be caused by the gap, but also by the flexible nature of the used mats, which differ from typical fixed base layers used in controlled laboratory conditions. Regardless, for a wide range of conditions and single meadow interaction, results showed that contemporary models can provide insight into the expected attenuation effects of the meadow, as long as uncertainties are also cross-examined and informed. Seagrass reestablishment efforts using ASG can thus be supported by existing flow-vegetation-interaction models by providing faithful magnitude projections of the effect of anchored mats in the field.

### Supplementary Information

Supplementary Information.

## Data Availability

The datasets used and analysed during the current study are available online at doi.org/10.25835/33om1uvt.

## List of symbols

$\alpha _w$: canopy attenuation parameter
$$\beta$$: damping coefficient
$\delta _{\textrm{PA}}$: density of polyamide
$\eta$: surface water level
$\hat{U}_{\infty ,w}^{\textrm{rms}}, \hat{U}_{w}^{\textrm{rms}}$: canopy integrated rms-velocities
$\hat{U}_w^*, U_{\infty ,w}^*, t^*$: non-dimensionalized parameters
$$\lambda$$: wavelength
$\lambda _f$: vegetation frontal area parameter
$\lambda _p$: vegetation plan area parameter
$\textrm{ADV}$: acoustic doppler velocimeter
$$\textrm{ASG}$$: artificial seagrass
$\textrm{M0, M1, M2}$: no-mat, 1-mat and 2-mat configurations
$$\textrm{PA}$$: polyamide
$\textrm{rms}$: root mean square
$$\textrm{USS}$$: ultrasonic sensor
$$\textrm{WR}$$: wave run
$$\nu$$: kinematic viscosity
$\omega$: wave angular frequency
$$\rho$$: water density
$$\varepsilon _D$$: energy dissipation
$$\varphi$$: wave phase
*a*: wave amplitude
$A_\infty ^{\textrm{rms}}$: rms free stream wave excursion length
$$b_1, b_2$$: fit constants
$b_v$: width of vegetation
$$C_D$$: drag coefficient
$$C_f$$: friction coefficient
$c_g$: wave group velocity
$$C_M$$: inertia coefficient
*Ca*: Cauchy number
*D*: depth to concrete flume bed
*d*: water depth over sand bed
$d_{50}$: median sediment particle size
*E*: energy density
*EI*: flexural rigidity
*g*: gravity constant
*H*: wave height
$H_0$: canopy-unaffected wave height
$h_c$: canopy height/upright vegetation length
*k*: wave number
$$k_m$$: added mass
$k_r, k_i$: real, imaginary components of wave number
*KC*: Keulegan-Carpenter number
*L*: relative velocity between blade and water
$L_d$: drag length scale
$l_e$: effective length of vegetation
$L_s$: shear length scale
*N*: areal density of vegetation
$n_{1-3}$: coefficients for the calculation of $C_D$
*Re*: Reynolds number
$Re^{l_e}$: effective-length-based Reynolds number
*S*: vegetation shoot separation
*T*: wave period
*t*: time
$$t_v$$: vegetation thickness
$$U'$$: turbulent velocity
*u*, *v*, *z*: velocity cardinal components
$U_b^{\textrm{max}}$: near-bed maximum velocity
$U_c$: steady flow velocity
$$U_i$$: instantaneous velocity
$$U_R$$: Ursell number
$U_w$: wave (oscillatory) velocity
$U_w^{\textrm{max}}$: maximum wave velocity
$U_w^{\textrm{min}}$: minimum wave velocity
$U_w^{\textrm{rms}}$: rms wave velocity
$U_{\infty ,w}^{\textrm{rms}}$: free stream rms wave velocity
*x*, *y*, *z*: coordinate system
