# Supplementary material for: Wave dynamics alteration by discontinuous flexible mats of artificial seagrass can support seagrass restoration efforts
Source: Sci Rep. 2023 Nov 8;13:19418. doi: 10.1038/s41598-023-46612-z (PMC10632504; doi:10.1038/s41598-023-46612-z)
Supplement: Supplementary file 1 — Supplementary Information. [file 41598_2023_46612_MOESM1_ESM.pdf]

SUPPORTING INFORMATION for:

Wave dynamics alteration by discontinuous flexible mats of artificial seagrass can support seagrass restoration efforts

Raúl Villanueva[1*]; Maike Paul[1] and Torsten Schlurmann[1]

[1] Leibniz University Hannover, Ludwig Franzius Institute for Hydraulic, Estuarine and Coastal Engineering, 30167, Hannover, Germany
[*]Corresponding author. Email: villanueva@lufi.uni-hannover.de

Studies of wave-vegetation interaction

The evolution of research on wave-vegetation interaction can be separated into studies dealing solely with wave attenuation and those focusing on the velocity structure. Originally, a Morison-based formula was used to calculate wave decay. This was related to the energy density of the waves and the loss of energy reflected through wave decay (Dalrymple et al. 1984). The loss of energy was expressed exponentially through the imaginary wave number k_i by relating the forces exerted by vegetation on flow. Linearization of this formulation led to the well-known damping coefficient β, used to calculate wave evolution as a function of the meadow length, i.e. wave decay. Wave decay is sometimes denoted as $K_v(x)$, where $K_v = 1/(1+\beta x)$, and x is the distance within the meadow following the direction of wave propagation.

With time, oscillatory flow and its effect on aquatic vegetation, which is usually flexible, was considered (Asano et al. 1992). Sway was taken into account through the relative velocity between the plant and the surrounding instantaneous flow component. This reduced drag, and thus wave damping. Calibration through the drag coefficient C_D thus began to vary. Chen et al. (2018) and Henry et al. (2015) provide a comprehensive overview and analysis of different C_D formulations based on either Re or KC. Equations S1–S5 represent common calibrated forms of C_D based on Re and KC resulting from empirical studies, where n_i are the fit coefficients, l_r is the reconfigured canopy height, and $KC_{l_r} = KC$ factored by l_r.

Furthermore, the characterization of Re and β using the effective length l_e became important as reconfiguration modifies vegetation effects on flow (Luhar et al. 2010; Losada et al. 2016). The considerations mentioned above are necessary to take into account plant traits to determine their effect on the velocity structure accurately.

Other studies focused on the changes in in-canopy velocities to describe vegetation-induced wave attenuation. Newer studies (e.g. Lei and Nepf 2019; Zhang et al. 2021) have incorporated separate drag calculations for the stem and blades of a plant shoot. Interaction among leaves has also been largely neglected. Zhang et al. (2021) include this from previous studies through a so-called sheltering coefficient.

$$C_D = n_1 + \left(\frac{n_2}{Re}\right)^{n_3} \tag{S1}$$

$$C_D = \frac{\exp\left(n_1 KC_{l_r}\right)}{KC_{l_r}^{n_2}} \tag{S2}$$

$$C_D = n_1 KC^{n_2} \tag{S3}$$

$$C_D = n_1 KC^{n_2} + n_3 \tag{S4}$$

$$C_D = \frac{n_1}{KC}^{n_2} + n_3 \tag{S5}$$

Table S1 shows a summary of relevant studies, ordered chronologically, to show the differences between experimental setups, hydrodynamic conditions trialed, vegetation characteristics, and the main focus of the studies.

Table S1.: Summary of wave-vegetation interaction studies

Study	Focus[b]	Exp. facility	Flume dim. (l[m]xb[m])	Waves tested	Wave Height H[cm]	Wave Period T[s]	Flow vel.[c] [cms^{-1}]	Vegetation[d]	Shoot density [m^{-2}]	Water Depth d[m]	Patch dim. (l[m]xb[m])	Canopy Height h_c[m]	Summary[a]
Asano et al. (1992)[e]	WD	flume	27x0.5	regular	5.6–12	0.71–2	-	Artificial Kelp (f)	1100/1490	0.45/0.52	8x0.5	0.25	$C_D = O(1)$, calibrated based on k_i, u_r
Fonseca and Cahalan (1992)	WD	flume	6.10x0.23	regular	1–20	0.6–3	-	live(4 species)	750–2870[g]	0.06–0.19[g]	1x0.23	0.17–0.41[g]	%-change of $E(x)$ as $f(d)$. Up to 40% reduction from seagrass
Dubi (1995)	WD	flume	33x1	regular + random	4.5–17	1.26–4.42	-	artificial (f)*L. hyperborea*	1200	0.4–1	9.3x0.5[h]	0.2	k_i governed by T, sensitive to d, N
Méndez et al. (1999)[e]	VS/ WD	flume	see [e]	regular + random	see [e]	see [e]	-	artificial kelp (f)	see [e]	see [e]	see [e]	see [e]	Re-based C_D for sway/no sway (Eq. S1). Reflection significant. H/H_0 function of drag forces and vegetation parameters
Mendez and Losada (2004)[e]	WD	flume	see [e]	random, breaking + non-breaking + shoaling	see [e]	see [e]	-	artificial kelp (f)	see [e]	see [e]	see [e]	see [e]	β model: KC-based C_D (Eq. S2, $KC_{l_r} = KC$ factored by l_r. No sway/reflection)
Lowe et al. (2005)	VS	flume	12x1.2	regular	-	1–3	2–5.3	cylinders (r)	100/ 44/25	0.43	1.8x1.2	0.1	α_w decreases with increasing A_∞
Lowe et al. (2007)	VS/ WD	field	-	random	-	2–25 (7.8 peak)	-	cylinders (r)	64	1.5–1.8	2.4x1.2	0.1	$\varepsilon = f(A_\infty)$, increases with increasing α_w. C_D scales as $C_D\lambda_f\alpha_w^3$
Bradley and Houser (2009)	WD	field	-	random	7–9	∼1–100 (0<freq[Hz]<1)	-	live *T. testudinum*	1100	1	43.3xInf	0.25–0.30	$k_i = f(T, E)$. New C_D based on Re (Eq. S1) and KC (Eq. S3)
Luhar et al. (2010)	VS	flume	24x0.38	regular	1.6–10.6	0.9–2	-	artificial (f) *Z. marina*	300–1800	0.16–0.39	5x0.38	0.13	mean current within canopy $f(A_\infty, x)$, attenuation characterized by α_w

continued on next page...

Table S1 Summary of wave-vegetation interaction studies (continued)

Study	Focus[b]	Exp. facility	Flume dim. (l[m]xb[m])	Waves tested	Wave Height H[cm]	Wave Period T[s]	Flow vel.[c] [cms^{-1}]	Vegetation[d]	Shoot density [m^{-2}]	Water Depth d[m]	Patch dim. (l[m]xb[m])	Canopy Height h_c[m]	Summary[a]
Stratigaki et al. (2011)	VS/ WD	flume	100x3	regular	39–43	2.3/3/ 3.5/4	-	artificial (f) *P. oceanica*	180/360	1.10/1.30/ 1.70	10.7x3	0.55	measured $H(x)/H_0$. Higher N, h_c/d = higher H reduction. u increases at canopy top
Paul et al. (2012)	WD	race-track flume	17.55x0.6	regular + current	10	1	10	artificial (f,r) *Z. noltii*	500–8000	0.3	3x0.6	0.10/ 0.15/ 0.30	measured $H(x)/H_0$ varying h_c, N, EI. Wave attenuation is $f(\lambda_f, \lambda_p, N)$. Current reduces wave attenuation
Pujol et al. (2013)	VS	flume	6x0.5	regular	-	0.714/ 1/1.25	-	cylinders (r), artif. (f) *P. Oceanica*	128/640/ 1280	0.3	2.5x0.5	0.14	Flow attenuation characterized by α_w. Velocity profile, wave-induced current dependent on h_c/d
Möller et al. (2014)	WD	flume	300x5	regular + random	0.1–0.9	1.5–6.2	-	real *Puccinellia*, *Elymus*	1225	2	39.44x5	220/ 700[g]	*Re*-based C_D formulation (Eq. S1) for regular, random waves
Hu et al. (2014)	WD	flume	40x0.8	regular + current	4–20	1–2.5	0–30	wooden rods (r)	62/ 139/ 556	0.25/ 0.5	6x0.8	0.36	*Re*-based C_D for combined waves+current fitted from β. U_c/U_w determine decay contribution of current
Ozeren et al. (2014)	WD	flume	20.6x0.69	regular + random	3–15	0.7–2	-	birch dowel (r), foam-cords (f) and real (2 species)	156/ 350/ 623/ 545/ 2857	0.5–0.7	3.66	0.48–1.03	C_D based on experimentally fitted β. Re, KC-based C_D formulations given (Eq. S1)
Losada et al. (2016)[f]	WD	3D wave basin	30x44	regular + random + current	12–20	1.2–2.2	30	live *P. maritima* and *S. anglica*	430 (*S.a.*)/ 877–1389 (*P.m.*)	0.4/ 0.6	6[j]	0.28/ 0.47	*Re*-based formulation of C_D (Eq. S1) for varying wave+current conditions

continued on next page...

Table S1 Summary of wave-vegetation interaction studies (continued)

Study	Focus[b]	Exp. facility	Flume dim. (l[m]xb[m])	Waves tested	Wave Height H[cm]	Wave Period T[s]	Flow vel.[c] [cms^{-1}]	Vegetation[d]	Shoot density [m^{-2}]	Water Depth d[m]	Patch dim. (l[m]xb[m])	Canopy Height h_c[m]	Summary[a]
El Allaoui et al. (2016)	VS	flume	6x0.5	regular	-	0.833	-	PE (f)	320/1280	0.3	0.84x0.5[i]	0.14	U_w, U' increase within gap, reduce for gap width $< 2h_c$ with N found to be important for shelter
Luhar et al. (2017)	WD	flume	24x0.38	regular	1.8–11.2	0.8–2	-	PE (f) around 2-cm dowel	300-1800	0.16–0.39	0.5x0.38	0.13	β better predicted by l_e, which depends on CaL. Link used to calculate ratio of bare bed and vegetated H
Abdolahpour et al. (2018)	VS	flume	50x1.2	regular	-	5–9	-	birch dowels (r), artif. (f) *P. australis*	400–3000	0.76	3–9x1.2	0.15/ 0.3	Vertical mixing quantified. Flexibility decreases shear, thus mixing, but increases wake velocities
Zhang et al. (2018)	VS	flume	24x0.38	regular	2–8.4	1/ 2	-	artificial (f) *Z. marina* and *V. americana*	280/ 600/ 820/ 1370	0.4–0.45	2/7x0.38	0.14	TKE is dependent on A_∞ and S, can be proportionally linked to ΔE (formulation proposed)
Chen et al. (2018)[k]	WD	flume	see [e]	regular + current	see [e]	see [e]	see [e]	wooden rods (r)	see [e]	see [e]	see [e]	see [e]	Obtaining C_D from force measurements vs. calibration through β. KC-based formulation of C_D (Eq. S4).
Lei and Nepf (2019)	WD	flume	24x0.38	regular	1.6–10	1/ 1.4/ 2	-	low-density PE (f) (t_v varies)	single blade + 280–1370	0.28	5x0.38	0.03/ 0.05/ 0.1/ 0.15	β calibration based on l_e scaling law (obtained from single blade experiments), good agreement for range of lab, field applications
van Veelen et al. (2020)	VS/ WD	flume	30.7x0.8	regular	10–20	1.4–2	-	bamboo dowel (r) and silicon (f)	1111	0.3–0.6	1.5x0.8	0.30	KC-based C_D from fitted β (Eq. S5), improved using in-canopy velocities

continued on next page...

Table S1 Summary of wave-vegetation interaction studies (continued)

Study	Focus[b]	Exp. facility	Flume dim. (l[m]xb[m])	Waves tested	Wave Height H[cm]	Wave Period T[s]	Flow vel.[c] [cms^{-1}]	Vegetation[d]	Shoot density [m^{-2}]	Water Depth d[m]	Patch dim. (l[m]xb[m])	Canopy Height h_c[m]	Summary[a]
van Rooijen et al. (2020)	VS	flume	35x1.2	regular	9–21	2–5	-	dowels (r)	3100	0.75	2.5x1.2	0.30	force-derived C_D applied to develop numerical model of vertical distribution of U_i in and above canopy
Zhang et al. (2021)	WD	flume	24x0.38	regular	2–8.2	1.1/ 1.4/ 2	-	artif. *S. alterniflora* (f)	280	0.18–0.45	4x0.38	0.30	validation of modified β based on scaling law CaL to account for differing morphological characteristics of single shoots
Schaefer and Nepf (2022)	WD	flume	24x0.38	regular	0(pure current)–8.2	2	2.7–10	artif. *Z. marina* low-density PE (f)	950	0.27/ 0.45	6.1x0.38	0.136	new β formulation for wave-current conditions based on l_e. l_e function of in-canopy velocity U_1 ($\neq U_c$)

Superscript legend:
[a] main outcome and approach taken, with E=energy density; ε=rate of energy dissipation (calculated based on forces); k_i=exponential decay coefficient; β=damping coefficient based on the linearized height evolution H/H_0; α_w=in-canopy flow attenuation; A_∞= wave excursion; u_r=relative velocity between plants and flow; l_r=reconfigured length due to flow; TKE=Turbulent Kinetic Energy. Other variables found in main text/nomenclature.
[b] VS: Velocity Structure; WD: Wave Decay
[c] for experiments with waves plus current
[d] (f): flexible, (r): rigid, PE=Polyethylene
[e] experimental data from Asano et al. (1988) for regular and Dubi (1995) for irregular
[f] details on experimental set-up in Lara et al. (2016) and Maza et al. (2015)
[g] species dependent
[h] flume partitioned
[i] with cross-sectional gaps
[j] circular patch of vegetation
[k] experimental data from Hu et al. (2014)

References

Abdolahpour, M., Ghisalberti, M., McMahon, K., and Lavery, P. S. (2018). The impact of flexibility on flow, turbulence, and vertical mixing in coastal canopies. *Limnology and Oceanography*, 63(6):2777–2792.

Asano, T., Deguchi, H., and Kobayashi, N. (1992). Interaction between water waves and vegetation. In *Coastal Engineering 1992*, pages 2709–2723. ASCE.

Asano, T., Tsutsui, S., and Sakai, T. (1988). Wave damping characteristics due to seaweed. In *Proc. 35th Conf. on Coastal Engrg., 1988.*

Bradley, K. and Houser, C. (2009). Relative velocity of seagrass blades: Implications for wave attenuation in low-energy environments. *Journal of Geophysical Research: Earth Surface*, 114(F1).

Chen, H., Ni, Y., Li, Y., Liu, F., Ou, S., Su, M., Peng, Y., Hu, Z., Uijttewaal, W., and Suzuki, T. (2018). Deriving vegetation drag coefficients in combined wave-current flows by calibration and direct measurement methods. *Advances in water resources*, 122:217–227.

Dalrymple, R. A., Kirby, J. T., and Hwang, P. A. (1984). Wave diffraction due to areas of energy dissipation. *Journal of waterway, port, coastal, and ocean engineering*, 110(1):67–79.

Dubi, A. (1995). *Damping of water waves by submerged vegetation: a case study on Laminaria hyperborea.* PhD thesis, Thesis, Department of Structural Engineering, The Norwegian Institute of Technology, Trondheim, Norway.

El Allaoui, N., Serra, T., Colomer, J., Soler, M., Casamitjana, X., and Oldham, C. (2016). Interactions between fragmented seagrass canopies and the local hydrodynamics. *PLoS One*, 11(5):e0156264.

Fonseca, M. S. and Cahalan, J. A. (1992). A preliminary evaluation of wave attenuation by four species of seagrass. *Estuarine, Coastal and Shelf Science*, 35(6):565–576.

Henry, P.-Y., Myrhaug, D., and Aberle, J. (2015). Drag forces on aquatic plants in nonlinear random waves plus current. *Estuarine, Coastal and Shelf Science*, 165:10–24.

Hu, Z., Suzuki, T., Zitman, T., Uittewaal, W., and Stive, M. (2014). Laboratory study on wave dissipation by vegetation in combined current–wave flow. *Coastal Engineering*, 88:131–142.

Lara, J. L., Maza, M., Ondiviela, B., Trinogga, J., Losada, I. J., Bouma, T. J., and Gordejuela, N. (2016). Large-scale 3-d experiments of wave and current interaction with real vegetation. part 1: Guidelines for physical modeling. *Coastal Engineering*, 107:70–83.

Lei, J. and Nepf, H. (2019). Wave damping by flexible vegetation: Connecting individual blade dynamics to the meadow scale. *Coastal Engineering*, 147:138–148.

Losada, I. J., Maza, M., and Lara, J. L. (2016). A new formulation for vegetation-induced damping under combined waves and currents. *Coastal Engineering*, 107:1–13.

Lowe, R. J., Falter, J. L., Koseff, J. R., Monismith, S. G., and Atkinson, M. J. (2007). Spectral wave flow attenuation within submerged canopies: Implications for wave energy dissipation. *Journal of Geophysical Research: Oceans*, 112(C5).

Lowe, R. J., Koseff, J. R., and Monismith, S. G. (2005). Oscillatory flow through submerged canopies: 1. velocity structure. *Journal of Geophysical Research: Oceans*, 110(C10).

Luhar, M., Coutu, S., Infantes, E., Fox, S., and Nepf, H. (2010). Wave-induced velocities inside a model seagrass bed. *Journal of Geophysical Research: Oceans*, 115(C12).

Luhar, M., Infantes, E., and Nepf, H. (2017). Seagrass blade motion under waves and its impact on wave decay. *Journal of Geophysical Research: Oceans*, 122(5):3736–3752.

Maza, M., Lara, J., Losada, I., Ondiviela, B., Trinogga, J., and Bouma, T. (2015). Large-scale 3-d experiments of wave and current interaction with real vegetation. part 2: Experimental analysis. *Coastal Engineering*, 106:73–86.

Mendez, F. J. and Losada, I. J. (2004). An empirical model to estimate the propagation of random breaking and nonbreaking waves over vegetation fields. *Coastal Engineering*, 51(2):103–118.

Méndez, F. J., Losada, I. J., and Losada, M. A. (1999). Hydrodynamics induced by wind waves in a vegetation field. *Journal of Geophysical Research: Oceans*, 104(C8):18383–18396.

Möller, I., Kudella, M., Rupprecht, F., Spencer, T., Paul, M., Van Wesenbeeck, B. K., Wolters, G., Jensen, K., Bouma, T. J., Miranda-Lange, M., et al. (2014). Wave attenuation over coastal salt marshes under storm surge conditions. *Nature Geoscience*, 7(10):727–731.

Ozeren, Y., Wren, D., and Wu, W. (2014). Experimental investigation of wave attenuation through model and live vegetation. *Journal of Waterway, Port, Coastal, and Ocean Engineering*,

140(5):04014019.
Paul, M., Bouma, T. J., and Amos, C. L. (2012). Wave attenuation by submerged vegetation: combining the effect of organism traits and tidal current. *Marine Ecology Progress Series*, 444:31–41.
Pujol, D., Serra, T., Colomer, J., and Casamitjana, X. (2013). Flow structure in canopy models dominated by progressive waves. *Journal of hydrology*, 486:281–292.
Schaefer, R. B. and Nepf, H. (2022). Wave damping by seagrass meadows in combined wave-current conditions. *Limnology and Oceanography*, 67(7):1554–1565.
Stratigaki, V., Manca, E., Prinos, P., Losada, I. J., Lara, J. L., Sclavo, M., Amos, C. L., Cáceres, I., and Sánchez-Arcilla, A. (2011). Large-scale experiments on wave propagation over *Posidonia oceanica*. *Journal of Hydraulic Research*, 49(sup1):31–43.
van Rooijen, A., Lowe, R., Rijnsdorp, D. P., Ghisalberti, M., Jacobsen, N. G., and McCall, R. (2020). Wave-driven mean flow dynamics in submerged canopies. *Journal of Geophysical Research: Oceans*, 125(3):e2019JC015935.
van Veelen, T. J., Fairchild, T. P., Reeve, D. E., and Karunarathna, H. (2020). Experimental study on vegetation flexibility as control parameter for wave damping and velocity structure. *Coastal Engineering*, 157:103648.
Zhang, X., Lin, P., and Nepf, H. (2021). A simple-wave damping model for flexible marsh plants. *Limnology and Oceanography*, 66(12):4182–4196.
Zhang, Y., Tang, C., and Nepf, H. (2018). Turbulent kinetic energy in submerged model canopies under oscillatory flow. *Water Resources Research*, 54(3):1734–1750.
